# Antiepileptic Drug Tiagabine Does Not Directly Target Key Cardiac Ion Channels Kv11.1, Nav1.5 and Cav1.2

**DOI:** 10.3390/molecules26123522

**Published:** 2021-06-09

**Authors:** Magdalena Kowalska, Łukasz Fijałkowski, Monika Kubacka, Kinga Sałat, Grzegorz Grześk, Jacek Nowaczyk, Alicja Nowaczyk

**Affiliations:** 1 Department of Organic Chemistry, Faculty of Pharmacy, Ludwik Rydygier Collegium Medicum in Bydgoszcz, Nicolaus Copernicus University in Toruń, 87-100 Toruń, Poland; magda.kowalska@doktorant.umk.pl (M.K.); l.fijalkowski@cm.umk.pl (Ł.F.); 2 Department of Pharmacodynamics, Chair of Pharmacodynamics, Jagiellonian University Medical College, 9 Medyczna St., 30-688 Krakow, Poland; monika.kubacka@uj.edu.pl (M.K.); kinga.salat@uj.edu.pl (K.S.); 3 Department of Cardiology and Clinical Pharmacology, Faculty of Health Sciences, Collegium Medicum in Bydgoszcz, Nicolaus Copernicus University, 75 Ujejskiego St., 85-168 Bydgoszcz, Poland; g.grzesk@cm.umk.pl; 4 Physical Chemistry and Chemistry of Polymers, Faculty of Chemistry, Nicolaus Copernicus University, 7 Gagarina St., 87-100 Toruń, Poland; jacek.nowaczyk@umk.pl

**Keywords:** tiagabine, cardiac voltage-gated ion channels, molecular modeling, ECG study

## Abstract

Tiagabine is an antiepileptic drug used for the treatment of partial seizures in humans. Recently, this drug has been found useful in several non-epileptic conditions, including anxiety, chronic pain and sleep disorders. Since tachycardia—an impairment of cardiac rhythm due to cardiac ion channel dysfunction—is one of the most commonly reported non-neurological adverse effects of this drug, in the present paper we have undertaken pharmacological and numerical studies to assess a potential cardiovascular risk associated with the use of tiagabine. A chemical interaction of tiagabine with a model of human voltage-gated ion channels (VGICs) is described using the molecular docking method. The obtained in silico results imply that the adverse effects reported so far in the clinical cardiological of tiagabine could not be directly attributed to its interactions with VGICs. This is also confirmed by the results from the isolated organ studies (i.e., calcium entry blocking properties test) and in vivo (electrocardiogram study) assays of the present research. It was found that tachycardia and other tiagabine-induced cardiac complications are not due to a direct effect of this drug on ventricular depolarization and repolarization.

## 1. Introduction

Epidemiological studies have consistently shown that people with epilepsy have a higher prevalence of structural cardiac disease than those without it [1]. The functioning of neurons, muscles and cardiac myocytes is based on action potentials (APs) generated by transmutational ion currents mediated mainly by sodium, calcium and potassium [2,3]. According to the guidelines of the Comprehensive in vitro Proarrhythmia Assay (CiPA), a set of six ion channels has been selected for which currents are important for both the repolarization and depolarization of the cardiac action potential (AP) [4]. There is some evidence, based on the effect of clinical drugs on cardiac APs, indicating the classification of cardiac ion channels into two classes [5,6,7]. The first class contains the most important cardiac ion channels, such as K_V_11.1, Na_V_1.5 and Ca_V_1.2. The second class comprises K_V_4.3, K_v_LQT1/mink and Kir2.1 and is less critical for the assessment of all drugs under CiPA [7,8,9].

A common feature of both neurological disorders (e.g., epilepsy and chronic pain) and cardiac dysrhythmias is cell (neuronal cell and cardiac myocyte, respectively) hyperexcitability [2]. Therefore, drugs affecting cell excitability threshold within the nervous tissue can also interact with cardiac cell APs and vice versa [10,11,12]. It has to be emphasized that this drug-induced effect might sometimes be harmful as it may lead to the occurrence of additional alterations in neuronal or cardiac cell reactivity, thus being a cause for additional drug-induced (iatrogenic) complications. From the safety pharmacology point of view, it is of key importance to recognize these potential risk factors as early as possible. The cardiac VGICs assay is an indispensable step and a high-quality assay must accompany any investigational new drug application. The in silico studies of drug binding to K_V_11.1, Na_V_1.5 and Ca_V_1.2 may be valuable assays for drugs and drug candidates at present [13]. 

Currently, several antiepileptic drugs were reported to have cardiotoxic and metabolic adverse effects [1,14]. Tiagabine (TGB) is an anticonvulsant drug used to treat partial seizures in humans. Recent results of clinical trials and animal studies indicate that it might be also effective in patients suffering from pain, insomnia or mood disorders and these activities are attributed to its inhibitory effect on GABA uptake [15,16]. TGB is a 96% protein-bound molecule [17]. The drug is a potent inhibitor of [3H]GABA uptake into synaptosomal membranes (IC_50_ = 67 nM) or neurons (IC_50_ = 446 nM) and glial cells (IC_50_ = 182 nM) in primary cell cultures. The in vivo tests have shown that TGB neuronal inhibiting is 2.5-fold more potent than glial GABA uptake [18,19,20]. The enhancement of GABA neurotransmission due to GABA transporter subtype 1 (GAT-1) inhibition might also explain most of adverse effects of TGB, including drowsiness, confusion and dizziness [18,21]. However, some other TGB-induced complications do not to be directly related to its influence on GABA concentration in the brain and other tissues.

Several recent reports have demonstrated that tachycardia is observed in about 1.0% of patients treated with TGB [18,22] but the mechanisms underlying this cardiotoxic effect are not known. In the literature there is a limited amount of data regarding the influence of TGB on cardiovascular functions and metabolism [23].

Since tachycardia is most frequently caused by impaired ion channel functions [24,25,26,27], in the in silico part of the present study a detailed analysis of the interactions between the human K_V_11.1, Na_V_1.5, Ca_V_1.2 and TGB was performed. In order to compare the strength of TGB’s binding to individual ion channels terfenadine (TEF) [28], batrachotoxin (BTX) [29,30] and nifedipine (NFD) [31] were selected as compounds strongly affecting these molecular targets. 

In the course of present study, in vivo tests in rats were performed. The in vivo assay comprised the assessment of TGB’s proarrhythmic potential and its effects on ECG components were studied; i.e., we conducted in vivo evaluation of TGB to assess its influence on PQ, QRS, QT and QTc intervals. Its effect on heart rate was also investigated. The relevant changes in PQ, QRS, QT and QTc intervals were interpreted as the effect of the test drug predominantly on Na_v_, K_v_ and Ca_v_ channels. In contrast, the changes in heart rate were treated as a measure of the effect of the test compound, particularly, on the Nav1.5 channel or autonomic system function. Calcium blocking properties of TGB were tested in the isolated rat aorta contracted with depolarizing KCl solution.

## 2. Results

### 2.1. Pharmacological Part

#### 2.1.1. The Effect on Normal Electrocardiogram

In vivo ECG study showed that TGB marked no significant effect on PQ, QRS, QT and QTc intervals. TGB also did not influence the heart rate significantly. It decreased the heart rhythm maximally at 30 min (by 6.9%) but this result did not reach statistical significance (Table 1). What is most important to note is that TGB at a dose as high as 100 mg/kg *i.p* did not prolong QT interval, which suggests that it did not prolong cardiac repolarization and probably did not block I_KR_ currents. Similar observations have also been made in the published studies [32,33]. We also did not observe the prolongation of PQ interval and QRS widening, which reflects the slowed conduction and disturbances in ventricular depolarization, usually due to the I_Na_ block. This is in line with the results obtained and presented here from molecular docking studies, where we found that TGB did not bind to cardiac voltage-gated ion channels K_v_11.1 (hERG) and Na_v_1.5.

#### 2.1.2. Voltage-Dependent Calcium Channels

**Figure 1:**
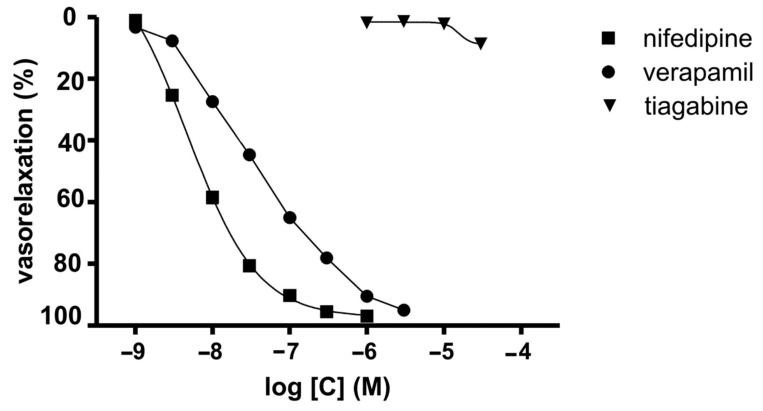
Inhibitory potencies of TGB and reference drugs (NFD and verapamil) on sustained contraction of aortic rings induced by KCl (60 mM).

During the course of our study, we investigated the calcium entry blocking properties of TGB in vasculature by employing the isolated rat aorta contracted with depolarizing KCl solution. In this experiment, the KCl-induced contraction was caused by an increase in extracellular potassium that leaded to membrane depolarization, which increases calcium influx from extracellular sources involving voltage-dependent calcium channels [34] (Ca_v_1.2). The reference compounds we used were verapamil [35] and NFD [36]. TGB was not able to relax KCl-precontracted aortic rings at the range of concentration 1–30 μM (Figure 1). At a higher concentration, TGB was not tested as it precipitated in Krebs–Henseleit solution. NFD, verapamil and voltage-dependent calcium channel blockers relaxed KCl (60 mmol/L)-precontracted aortic rings in a dose-dependent manner (Figure 1) by 95–97%, with the IC_50_ values of 4.7 ± 0.2 nM and 32.9 ± 7.4 nM, respectively [35,36]. On the basis of these results, we may state that TGB does not possess voltage-dependent calcium channel blocking properties at the tested range of concentrations. 

### 2.2. Molecular Docking Studies

#### 2.2.1. Terfenadine

**Figure 2:**
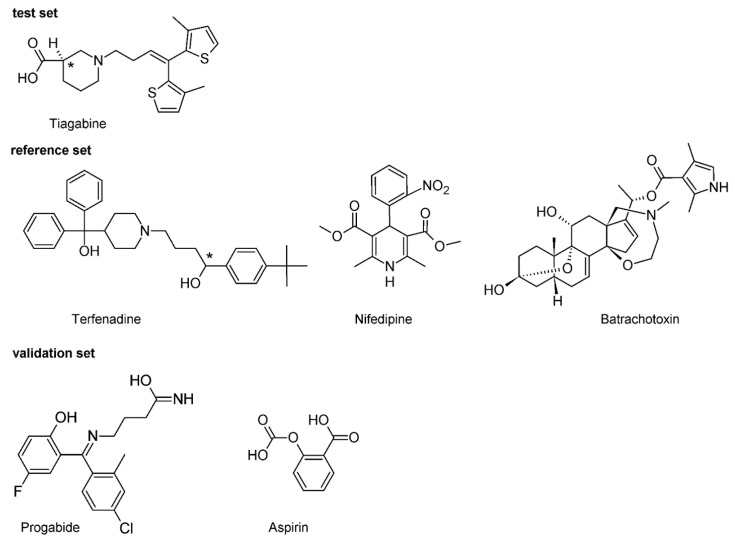
Chemical structure of the investigated compounds. Carbons at a tetrahedral stereogenic center are distinguished by *.

TEF is (RS)-1-(4-tert-butylphenyl)-4-{4-[hydroxy(diphenyl)methyl]piperidin-1-yl}-butan-1-ol and, from a chemical point of view, belongs to piperidine derivatives (Figure 2). 

The obtained results from molecular docking confirmed the strongest blocking effects of TEF on the hK_v_11.1 channel. R-TEF-hK_v_11.1 is the most stable complex in the studied set. Comparison of the data (such as E_B_ and pK_i_, Table 2) obtained for R-TEF and S-TEF complexes leads to the conclusion that there are significant stereoselective differences in the potential interaction for all studied channels. Docking experimentation predicted more effective potential interactions of all studied channels and S-TEF than its counterpart R-TEF (Table 2). Nevertheless, all calculated h-bonds formed are weak interactions. Moreover, obtained data suggests that R/S-TEF displays non inhibitory effects for hCa_v_1.2. According to data in Table 2, pKi < 3.5, while by convention pK_i_ ≤ 4 indicates the lack of a biological effect. Based on the CiPA studies, including the examination of the effect of 30 clinical drugs on the 7-ion channel [7], it can be concluded that the risk level of torsade de pointes (TdP) is correlated with the blocking effects of hK_v_11.1, hNa_v_1.5 and hCa_v_1.2. Crumb et al. [7,37] in their research proposed a classification of drugs into three categories of TdP entry risk (high, medium and low). According to their studies, drugs belonging to the high and medium risk stand out with block hERG to a much greater extent than any other tested currents. The drugs belonging to the low risk category is distinct with the non-specific blocking of hK_v_11.1, hNa_v_1.5 and hCa_v_1.2 channels (Figure S1). These results clearly indicate the need for testing drug candidates on the ion channel panel. In our study it was found, on the molecular level, that the high risk of TdP is a result of TEF’s strong blocking effects on hERG. R-TEF-hK_v_11.1 complex has one normal h-bond in which the hydroxyl group of the (4-tert-butylphenyl)methanol moieties of R-TEF donates energetically weak (−3.62 kcal/mol), short (2.12 Å) and almost linear interactions (158°) to Tyr652 (Figure S2). Regarding the interaction of R-TEF and S-TEF with the hNa_v_1.5 channel, it should be noted that both enantiomers of TEF practically strongly interact with this protein (pK_i_ > 5, Table 2), which also indicates their arrhythmogenic effects through the hNa_v_1.5. This observation is also confirmed by previously presented pharmacological studies [38]. 

#### 2.2.2. Nifedipine

NFD is a 3,5-dimethyl 2.6-dimethyl-4-(2-nitrophenyl)-1.4-dihydropyridine-3,5-dicarboxylate (Figure 2). It is classified as a dihydropyridine subclass compound. It is a highly apolar photosensitive compound. The NFD docking experiment revealed that this molecule can interact with an active site of all the studied proteins (Table 2). It can be set to the following descending order of binding energies E_B(NFD-hKv11.1)_ ≈ −4.42, E_B(NFD-hNav1.5)_ ≈ −7.00 and E_B(NFD-hCav1.2)_ ≈ −10.71 kcal/mol, respectively. The binding energies obtained in the docking experiment show that NFD forms a more stable complex in the case of the hCa_v_1.2 channel. The in silico data obtained for blocking of hCa_v_1.2 channel (pK_i_ = 7.85) are in line with data in the literature (pIC_50_ = 7.48) [35] and (pK_i_ = 7.66) [39]. Based on the predicted binding affinity, the highest affinity was observed with the hCa_v_1.2 and NFD in all cases of studied channels. Additionally, the blocking effect of NFD with hNa_v_1.5 was also proven (Table 2). It has to be highlighted that in NFD-hNa_v_1.5 and NFD-hK_v_11.1 complexes, NFD interacts via one normal h-bond. In contrast, the complex NFD-hCa_v_1.2 shows two h-bonds, both of which are short (≈1.68 Å) and strong (≈−3.8 kcal/mol) (Table 2.). The assessment of the data obtained for the hydrogen bonds clearly leads to the conclusion that they all have incomparable energies and bond lengths. Interestingly, NFD forms the strongest h-bond with hK_v_11.1 and the weakest one with hNa_v_1.5 channel (Table 2. Figure S2). The h-bond with hCa_v_1.2 is characterized by an indirect force of influence. However, taking into account the data from Table 2, there is no correlation between the binding affinities of NFD and h-bond energy. The obtained distribution of estimated pK_i_ measure shows that in case of the hK_v_11.1 channel, NFD has no inhibitory effect for this channel (pK_i_ < 4. Table 2). These data prove that NFD has potency only in inhibiting the hCa_v_1.2 and hNa_v_1.5 channel, which is in agreement with the pharmacological data previously presented in pharmacological literature [40]. 

#### 2.2.3. Batrachotoxin

BTX as a steroidal alkaloid belongs to class A channel opening toxins [41]. Its molecule contains an oxazepane ring with tertiary amine and an aromatic pyrrole ring connected to the rigid polycyclic steroidal core via the ester group (Figure 2). Its 3D structure adopts a horseshoe conformation [42,43]. The outer surface of the horseshoe is hydrophobic, while the inner one is rather hydrophilic and forms the oxygen triad (at C3, C9 and C11) [44,45]. The outer surface of the horseshoe is hydrophobic, while the inner one is rather hydrophilic and forms oxygen triad (at C3, C9 and C11) [44]. It was suggested in the literature that this oxygen triad forms a hydrophilic arc, which can be regarded as a chelating site attracting some cations [46]. In the preliminary analysis of the docking, it was observed that BTX interacts with the active site of all studied proteins (Figure S2). The achieved data demonstrated that Na_v_1.5 forms one h-bond, while hCa_v_1.2 and hK_v_11.1 form two h-bonds. The energies obtained in the present in silico experiment show that hCa_v_1.2 and hK_v_11.1 form more and form stronger hydrogen bonds than the remaining one (hNa_v_1.5). The predicted binding affinity can be arranged in the following increasing order: hK_v_11.1 < hCa_v_1.2 < hNa_v_1.5 (Table 2). Obtained results revealed that BTX-hNa_v_1.5 is an energetically more stable complex with a binding energy of −9.01 kcal/mol. The stability of the complex with the hCa_v_1.2 and hK_v_11.1 is slightly lower than that with hNa_v_1.5 (i.e., E_B BTX-hCav1.2_ = −7.17 and E_B BTX-hKv11.1_ = −6.75 kcal/mol, respectively). The calculated BTX affinity value of pKi = 6.68 is consistent with the relevant inhibitory potential data known from the specialized literature (pIC_50_ = 6.71) [47]. The BTX-hNa_v_1.5 complex is formed via a single key interaction with residues of hNa_v_1.5. The hydroxyl group at position C-11 of the steroid skeleton donates one h-bond to the sulfur atom of Ser1458. This interaction is non-linear (157°) and with weak energy, i.e., −3.43 kcal/mol and a small bond length equal to 2.18 Å. This is in line with the literature data according to which the atom included in oxygen triad is responsible for the toxic interaction of BTX with its molecular target [44]. We can treat these data as a strong argument proving that BTX acts on the cytoplasmic side of the channel just as other Class A neurotoxic compounds do, as it is shown in Figure S2.

For hCa_v_1.2 and hK_v_11.1 channels, BTX has pK_i_ ≈ 5.2, which suggests comparable blocking effects. The BTX-hCa_v_1.2 complex is a more energetically stable form than the BTX-hNa_v_1.5 complex (Table 2). This complex has one normal h-bond. In the bifurcated h-bond, the major component is Ser1132 and the minor component is Gln1060; the associated energies are weak (E_HB_ = −0.37 kcal/mol and −3.23 kcal/mol, respectively). The distances of the hydrogen bonds lay in the range from 1.69 to 2.22 Å and, due to this, the three-centered hydrogen bond is highly not symmetric. In the case of BTX-hCa_v_1.2 complex, it is formed via one normal h-bond interaction (Table 2). 

#### 2.2.4. Tiagabine

**Figure 3:**
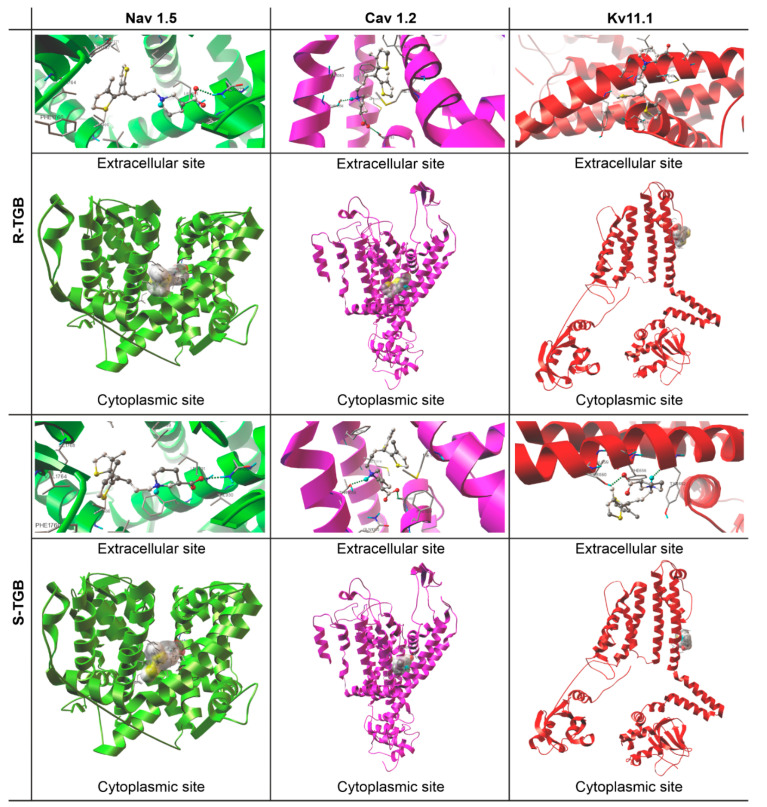
Pocket locations and binding modes of (R/S)-Tiagabine (R/S-TGB) and the investigated channels: hNav1.5, hCav1.2 and hKv11.1. Ligands (ball and stick model) and calculated hydrogen bonds (dashed green lines).

As it can be seen from the data collected in Table 2, TGB complexes between hNa_v_1.5, hCa_v_1.2 and hK_v_11.1 have a calculated pK_i_ ≤ 4, which is commonly used as a threshold and this drug can thus be considered as an inactive ligand for those proteins. These data refer to both R/S enantiomers of TGB (Figure 3). Docking TGB into the hNa_v_1.5 channel showed the highest pK_i_ value in Table 2, however, the value is still below the activity threshold level of pK_i_ > 4. The lowest pK_i_ was observed for hK_v_11.1. We can treat these data as a partial explanation of the reported adverse TGB interactions in the cardiovascular system. It also seems that a small percentage of the observed cardiac disorders can be attributed to the fact that TGB does not interact with hKv11.1, for which inhibition is responsible for QT_c_ prolongation. In all analyzed cases, R-TGB shows slightly higher binding affinities than S-TGB. This observation is in line with many previous pharmacological studies indicating the greater biological activity of R enantiomers compared to the S ones [10,11,12]. It is also worth emphasizing that all h-bonds formed between hCa_v_1.2 channels and R/S-TGB possess very favorable key interaction energy values (E_HB_ ≈ −7.32 for hCa_v_1.2-R-TGB and −5.54 kcal/mol for hCa_v_1.2-S-TGB, Table 2) and geometrically nonlinear systems. 

To sum up, the docking experiment revealed that R/S-TGB has lower intermolecular forces with all studied ion channels (E_B_ ≈ −5.30 kcal/mol, Table 2). The combination of the above-mentioned docking data with pharmacological as well as literature data regarding the risk for tachycardia due to TGB administration suggests that this adverse effect observed in humans is not likely to result from TGB interaction with molecular anti-targets (i.e., ion channels tested in this study) used for the cardiac risk assessment. The data obtained in this study allowed the supplementation of information on the impact of TGB on other than GAT-1 molecular targets. As is currently known, TGB has no significant affinity to other uptake systems, such as those for dopamine, noradrenaline, acetylcholine, adenosine, serotonin, histamine, opiate, glycine, glutamate or GABA [48,49]. It only has a weak affinity towards benzodiazepine receptors and does not affect K^+^ and Ca^2+^, while it slightly affects Na^+^ and cardiovascular channel function [19]. 

### 2.3. Validation Experiment

The validation was carried out by the docking of molecules with no affinity to hNa_v_1.5, hCa_v_1.2 and hK_v_11.1 channels, such as progabide (PRG) [50,51] and acetylsalicylic acid (ASA) [52]. This choice of PRG and ASA was made based on their chemical and biological similarity to TGB (ATC code: N03AG06 [53]). PRG (ATC code: N03AG05 [53]) is a first-generation antiepileptic drug without analgesic properties [12,54]. ASA (ATC code: N02BA01 [52]) is a classical and peripherally-acting nonsteroidal anti-inflammatory drug that is inactive at GAT and recommended for the prevention of several cardiovascular diseases due to its antiplatelet activity [52].

The results of the validation experiments, such as the complex binding energies, the specific hydrogen bond components and detailed data of the hydrogen bond features (energies, lengths and angles) are gathered in Table 3. The binding modes between hNa_v_1.5, hCa_v_1.2 and hK_v_11.1; and the control compound are illustrated in Figure S3 in the supplementary file. As it can be observed from the data, all control complexes have a calculated pK_i_ < 4, which is commonly used as the threshold and therefore the test compounds can be considered to be inactive ligands for those proteins. In addition, all control complexes have higher binding energy values and lower hydrogen bond energy than the TGB-hVGICs complex.

## 3. Discussion

Taking into consideration the new action profile of drugs already on the market, pharmacological safety is a particularly important issue. The main reason for this is the fact that for drugs with an extended therapeutic range (i.e., repurposed medications), the number of patients for whom a given drug is recommended will increase significantly. TGB discussed in this study belongs to this group of drugs. TGB is an anticonvulsant medication. It is also used in the treatment of anxiety-related disorders, as are a few other anticonvulsants [21]. In the case of this drug, the above mentioned point is particularly important because TGB was originally prescribed to a relatively small group of patients due to its narrow range of indicators (adjunctive treatment of partial seizures in adults and children 12 years of age and older) [56]. However, when we consider its analgesic and anxiolytic effects in our considerations, we include therapeutic indications for a significantly larger group of patients. Reports presented in the scientific literature indicate an approximate 70% increase in the number of patients on TGB therapy [57]. This is all the more important as the safety and efficacy of TGB have not been systematically evaluated for indications other than epilepsy.

Modern technology provides us with many different possibilities, the use of which should create the conditions to learn about safety pharmacology. Undoubtedly, in silico research is one of many possibilities for seeking answers in this regard. It seems to us that the research presented above makes some important contributions to this issue. The study tried to answer the question about the cardiovascular safety assessment of TGB. This is all the more important in the light of the recent expert discussions focused on extending the pharmacological profile of TBG. Many modern studies indicate that, in addition to the therapeutic use of TGB in epilepsy, we should strongly consider its utilization for non-epileptic indications. On the other hand, it is known that drugs that show this type of biological activity might have a strong effect on a heart. This, in turn, undoubtedly forces us to increase the effort focused on assessing cardiac safety. Considering the assessment of the effect of the compound on so-called anti-targets adopted from CiPA in the cardiac risk assessment, the TGB interaction was investigated with the following channels: hK_v_11.1, hNa_v_1.5 and hCa_v_1.2. Drugs strongly affecting individual channels (such as TEF, BTX and NFD) were selected as reference compounds for this study. TEF is a prodrug metabolized by intestinal CYP3A4 to fexofenadine, the active form being a selective histamine H1-receptor antagonist with antihistaminic and non-sedative effects. As it is well known, antihistamines may increase the rate of heart beat [58,59]. TEF causes prolonged repolarization, as is reflected in the broadening of the electrocardiographic QT interval, with the potential for serious ventricular arrhythmia and death [60,61,62]. Due to this, in the U.S. TEF was superseded by its active metabolite fexofenadine in the 1990s [59]. Numerous studies have proven that the ability of TEF to extend the QT interval depends on its binding to the Kv11.1 (Figure S1) protein encoded by hERG [63,64]. Nevertheless, TEF does not readily cross the blood–brain barrier and due to this its CNS, depression is minimal. NFD is a calcium channel blocker, a specific antagonist of Cav1.2 channels [65]. It is used to treat hypertension and chronic stable angina. NFD binds directly to inactive calcium channels and stabilizes their inactive conformation. By inhibiting the influx of calcium in smooth muscle cells, NFD prevents calcium-dependent myocyte contraction and vasoconstriction [31,40]. BTX was chosen as a reference compound in our docking experiment study due to its extremely potent cardiotoxic and neurotoxic characteristics [29,42,46,47,66]. In animals, BTX inactivates sodium channels in nerve cells and muscle cells, thereby interfering with the electrical signals sent throughout the body and causing fibrillation, arrhythmias, cardiac failure and death [29]. It is worth emphasizing that the obtained data from molecular modeling confirmed the high selectivity of the reference compounds for the appropriate ion channels (Table 2). The response obtained from molecular studies also indicates that the mechanisms underlying tachycardia in patients treated with TGB appear to be unrelated to its effect on the hNa_v_1.5, hCa_v_1.2 and hK_v_11.1 heart ion channels. Moreover, it is known that one of the most common mechanisms of drug-induced ventricular tachycardia is the blocking of hERG channels [67,68]. For this reason, additional evidence supporting the conclusions of the in silico study appears to provide epidemiological data (pharmacological reports) in which tachycardia is noted in approximately 1.0% of patients treated with TGB. In light of these facts, if TGB-induced tachycardia contacts the hK_v_11.1, hNa_v_1.5 and hCa_v_1.2. blocking mechanism, one would expect a higher rate of these side effects. In addition to the above-mentioned in vivo tests, as a part of safety pharmacology experiments, the TGB effects on PQ, QRS and QT intervals and the effects of TGB on heart rate were assessed. Many years of research have demonstrated that proarrhythmic effect, QT prolongation and hERG blocking cannot be treated as the only determinants of the occurrence of TdP. For instance, verapamil and ranolazines are examples of drugs that are strong inhibitors of the hERG channel and are simultaneously devoid of the risk of inducing arrhythmias and, vice versa, devoid of serious disorders of cardiomyocyte electrophysiology caused by drugs that are weak hERG inhibitors (e.g., sotalol and alfuzosin) [24,69]. Thus, this proves the insufficient specificity of the tests based only on the assessment of the hERG channel blocking potential. The risk of drug-induced TdP is rather balanced by multiple internal cardiac ionic currents that define ventricular repolarization. Therefore, studies that utilize the whole tissue seem to be a good option for the reflection of pharmacodynamics and potential adverse effects of a drug in a living organism. The in vivo results obtained from studies in rats showed that TGB did not prolong the QT interval or alter the QRS complex which suggests that it did not affect ventricular depolarization and repolarization. Taking into account that as in many other animal models of human diseases and also in this particular rat model, there might be basic translational problems. Some fundamental differences in the cardiac electrophysiology and myocyte calcium/potassium handling between rodents and humans have been suggested [70], but, nonetheless, the ECG in rats is still a widely applied experimental method in basic cardiovascular research. The technique of ECG recordings is simple; however, the interpretation of electrocardiographic parameters might be challenging. This is because the analysis may be biased by experimental settings, such as the type of anesthesia and the strain or age of animals. Furthermore, differences and similarities between rat and human ECG are frequently discussed in the context of translational cardiovascular research. Despite this, rat electrocardiography is an important investigational tool in experimental cardiology, even if the interpretation of electrocardiographic parameters is problematic [71]. In addition to this, a number of studies have shown that cardiotoxic drugs prolong QT interval in rodents and ECG recordings in rats have been used as a screening tool to assess the cardiotoxicity of various drugs. However, it needs to be stressed that the translation of the results of those studies to human application also possesses limitations. This is because rats’ hearts do not express hERG, whereas the cardiotoxicity of drugs is strongly associated with the blockade of hERG-related potassium channels. However, rat hearts express a variant of Ether-à-go-go-Related Gene (rat ERG, also known as Kcnh2) [72,73], which may also play a key role in the assessment of drug-induced cardiotoxicity. Taken together, we are aware that extrapolating the results from our rat model to humans should be performed extremely cautiously and this, of course, should be regarded as the main limitation of our study. Therefore, one can assume that its direct interaction with heart sodium and potassium channels can be neglected. TGB also marks no significant effect on the PQ interval, which suggests that it does not influence the atrio-ventricular conduction time. Consequently, we can postulate that TGB has low pro-arrhythmic potential, at least, after a single administration. Given that tachycardia may result due to a number of different mechanisms and not all of them directly affect ion channels, it is also necessary to evaluate the effects of TGB on several neurotransmitters/neuromodulators and the activity of the autonomic nervous system. However, these effects were not investigated in the present research. Further studies are necessary to assess the effect of TGB on the cardiovascular system, especially after chronic administration. On the basis of our research, we can state that TGB did not bind to voltage-gated ion channels and did not affect them directly. Furthermore, the observed accidents of tachycardia are probably not due to the direct effect of TGB on voltage-gated ion channels.

## 4. Methods

### 4.1. Pharmacological Studies General Information

The experiments were carried out using male Wistar rats (Krf:(WI) (WU), 200–250 g). The animals were housed in constant temperature facilities exposed to 12:12 h light/dark cycles and were maintained on a standard pellet diet with tap water given ad libitum. All procedures were conducted according to guidelines of ICLAS (International Council on Laboratory Animal Science) and approved by the Second Local Ethics Committee in Krakow, Poland (resolution No. 106/2016, 14 June 2016).

#### 4.1.1. Voltage-Dependent Calcium Channels—Functional Assays

In order to investigate the calcium entry blocking properties of TGB, it was tested on isolated rat aorta precontracted with KCl. Rats were anaesthetized with thiopental sodium (75 mg/kg, i.p., Rotexmedica, Germany) and the thoracic aorta was dissected, cleaned, denuded of endothelium, cut and mounted as described earlier [35]. Briefly, aorta rings were incubated in 30 mL chambers filled with a Krebs–Henseleit solution (NaCl 118 mM, KCl4.7 mM, CaCl_2_ 2.25 mM, MgSO_4_ 1.64 mM, KH_2_PO_4_ 1.18 mM, NaHCO_3_ 24.88 mM, glucose 10 mM, C_3_H_3_O_3_Na 2.2 mM and EDTA 0.05 mM) at 37 °C and pH 7.4 with constant oxygenation (O_2_/CO_2_, 19:1) and connected to an isometric FDT10-A force displacement transducer (BIOPAC Systems, Inc., COMMAT Ltd., Ankara, Turkey). The aortic rings were stretched and maintained at an optimal tension of 2 g and permitted to equilibrate for 2 h. The aortic rings were contracted to submaximal tension with KCl (60 mmol/L). Once the plateau was attained, concentration-relaxation curves were obtained by the addition of cumulative doses of tiagabine to the precontracted preparations. 

Concentration-response curves were analyzed using GraphPad Prism 5.0 software (GraphPad Software Inc., San Diego, CA, USA). Relaxations are expressed as a percentage of inhibition of the maximal tension obtained with the contractile agent (Emax = 100%). Data are the means ± SEM of at least 4 separate experiments. 

#### 4.1.2. The Effect on Normal Electrocardiogram

In vivo electrocardiographic investigations were carried out using an ASPEL ASCARD B5 apparatus (Aspel, Poland), standard lead II and paper speed of 50 mm/s. The ECG was recorded just prior to and also at 1, 5, 10, 20, 30, 40, 50 and 60 min following the i.p. administration of TGB at a dose of 100 mg/kg. The QT_c_ was calculated according to the formula of Bazzett: QT_c_ = QT/√RR [74]

### 4.2. In Silico Studies

The calculation procedures applied in the study are typical for the processing of docking studies. 

#### 4.2.1. Ligand Preparation

For the 3D molecular structure calculations, the Gaussian 09 (version D.01. for Unix/Linux) package was used [75]. The initial acceptable 3D structures of 6 studied compounds (Figure 2) were downloaded (as mol2 file) from ZINC [76]. Later, the GaussView [75,77] was applied for preparation of Gaussian input files. All the molecules were geometry-optimized in water as described by the PCM (polarizable continuum model). DFT/B3LYP level of theory 6311 + G(d, p) basis set was used. After geometrical optimization, (the root-mean-square gradient value smaller than 10^−6^ a.u.) compounds were saved as mol2 files using the GaussView. Subsequently, torsionals and the number of active torsions for ligands were defined and the Gasteiger charges were assigned to each compound via AutoDockTools (ADT) [78]. Finally, ligands prepared for docking were saved as pdbqt files.

#### 4.2.2. Voltage-Gated Ion Channels Preparation

- hNa_v_1.5 preparation:

The lack of crystal structure of hNa_V_1.5 pore domain causes the need for preparing homology 3D models for this protein. For our research, the sequence for the hNa_v_1.5 protein was gained from the Swiss Model Repository (SMR). SMR is a database which currently holds over 400,000 high quality 3D protein structure models generated by the automated SWISS-MODEL homology modeling pipeline [79]. The pdb file was downloaded from SWISS-MODEL SERVER (accession number Q14524) [80]. For this alignment, X-ray structure of human Na_v_1.2-β2-KIIIA ternary complex (PDB entry 6J8E) was employed. Sequence identity between template and the monomer of sodium channel protein type 5 subunit α is 66.70%. Subsequently, the pdb file was opened in ADT [78]. ADT read coordinates, added charges, merged non-polar hydrogens and assigned appropriate atom types. Before formatting a molecule for AutoDock, we removed 9Z9 ((3β,14β,17β,25R)-3-[4-methoxy-3-(methoxymethyl)-butoxyl]-spirost-5-en), which is irrelevant molecule in this experiment. Finally, the prepared protein was saved as a pdbqt file. In the computational part of the study, we pondered the interaction between the investigated ligands and the intracellular pore gate formed from the proper residues of chain A.

- hCa_v_1.2 preparation

The dearth of a 3D structure of hCa_v_1.2 proper region also causes need for preparing homology models of this protein. The sequence for the hCa_v_1.2 was gained from the SMR as well (accession number Q13936). For this alignment, X-ray structures of nifedipine complex with rabbit Ca_v_1.1 (PDB entry 6JP5) were employed. The sequence identity between template and isoform 4 of CAC1C_HUMAN Voltage-dependent L-type calcium channel subunit alpha-1C is 70.31% and, according to the best of our knowledge, this is one of the highest identities currently available. Subsequently, similar to the hNav1.5 protein, the pdb file was opened in ADT [58]. The next steps were also analogous. Before the docking experiment, we removed C8U (methyl (4~{S})-2,6-dimethyl-5-nitro-4-[2-(trifluoromethyl)phenyl]-1,4-dihydropyridine-3-carboxylate)), which is a pointless ligand in this case. The pore forming and dihydropiridyne binding residues (from ARG1109 to LYS1198) were considered as the ligand binding site [81].

- hK_v_11.1 preparation:

The sequence for the potassium voltage-gated channel subfamily H member 2 protein was downloaded from the Research Collaboratory for Structural Bioinformatics (RCSB) Protein Data Bank (PDB entry 5va2) as the crystal structure [82]. As in previous proteins, the pdb file was opened in ADT, read coordinates, added charges, merged non-polar hydrogens and assigned the appropriate atom types. As usual, we also removed crystallographic waters from 5va2. The binding pocket of the studied molecule were composed of the pore forming segment H5 and transmembrane helical fragment-Segment S6 [83].

#### 4.2.3. Molecular Docking

Molecular docking was performed using the AutoDockTools 4.2 suite of the program [55]. A grid box with a dimension of 60 × 60 × 60 Å^3^ and grid spacing of 0.375 Å, which is large enough for a free rotation of a ligands, was built in the middle of the binding pockets of the studied Voltage Gated Ion Channels (VGICs) channels, which are composed using the appropriate residues (Table 4).

Torsionals in the residuals of the binding pocket were not rotatable. The rigid docking was carried out using the Lamarckian genetic algorithm 4. The optimized docking parameters were set as default values, with the exception of the number of genetic algorithms run which was 100. Torsionals in the ligands were rotatable-6 active torsions in each ligand (except for terfenadine, where it was 11). A cluster analysis was performed using RMS tolerance of 2 Å. In each case, the best docking result was considered as the complex with the lowest binding energy. Interactions between ligands and the related channel models were analyzed using the AutoDockTools program (ADT. Version 1.5.4) [78].

## Figures and Tables

**Table 1:** Effects of TGB (100 mg/kg *i.p.*) on the heart rate and ECG intervals in anesthetized rat (thiopental 75 mg/kg *i.p.*).

Parameters	Time of Observation (min)							
	0	5	10	20	30	40	50	60
Beats/min	288.0 ± 5.4	275.6 ± 7.6	274.2 ± 7.7	269.5 ± 6.6	268.1 ± 3.0	276.7 ± 4.7	283.7 ± 7.0	289.5 ± 11.3
PQ (ms)	52.6 ± 2.0	56.2 ± 2.0	56.8 ± 2.1	56.6 ± 18	57.2 ± 1.3	55.2 ± 2.2	57.2 ± 1.2	56.8 ± 1.3
QRS (ms)	19.2 ± 0.5	18.4 ± 1.1	19.8 ± 0.9	19.4 ± 0.6	19.4 ± 0.9	19.8 ± 0.5	19.6 ± 0.4	19.0 ± 0.9
QT (ms)	61.0 ± 1.0	60.6 ± 0.4	62.6 ± 1.9	62.8 ± 1.8	60.8 ± 0.8	63.6 ± 1.8	61.2 ± 0.8	61.6 ± 0.9
QTc (ms)	113.8 ± 3.0	115.6 ± 1.5	119.9 ±5.6	121.1 ±3.8	117.4 ± 2.0	121.0 ± 3.8	115.0 ± 2.0	114.8± 3.0

The data are the means of five experiments ± S.E.M. Statistical analysis: one-way analysis of variance (ANOVA) with repeated measurements and followed by Dunnett’s post hoc test.

**Table 2:** The summary of hNa_v_1.5, hCa_v_1.2 and hK_v_11.1 (hERG) channel docking experiment results.

Complex		E_B_	pK_i_	Amino Acid Residues	H_B_		Angle	L_HB_	E_HB_
Protein	Ligand	kcal/mol			donor	acc	θ	Å	kcal/mol
hNa_v_1.5	R-TGB	−5.01	3.74	ASN927	#CONH_2_	%COO	153.33	2.17	−3.38
	S-TGB	−5.21	3.82	ASN927	#CONH_2_	%COO	171.54	2.23	−4.05
	NFD	−7.00	5.13	LEU409	%NOH	#CONH	146.945	1.987	−0.07
	R-TEF	−6,83	5.01	none					
	S-TEF	−7.45	5.46	LEU409	%OH	#CONH	163.079	2.014	−1.00
				GLU417	%OH	#COO	129.495	2.178	−0.04
	BTX	−9.01	6.68	SER1458	%NH	#CONH	156.904	2.179	−3.43
hCa_v_1.2	R-TGB	−5.05	3.70	THR1056	%NH	#OH	168.494	1.868	−7.33
				SER1132	#OH	%COO	176.604	2.116	−5.54
	S-TGB	−4.77	3.50	THR1056	%NH	#OH	150.676	2.155	−4.06
				SER1132	#OH	%COO	137.882	2.092	−0.01
	NFD	−10.71	7.85	THR1462	%NOH	#CONH	124.62	1.68	−3.13
				TYR1508	#PhOH	%NOH	141.07	1.92	−3.84
	R-TEF	−4.16	3.05	THR1133	%OH	#CONH	137.428	2.021	−0.35
	S-TEF	−4.75	3.48	ALA1174	%OH	#CONH	128.006	1.901	−0.35
	BTX	−7.17	5.25	GLN1060	%OH	#CONH2	157.582	1.691	−0.37
				SER1132	#OH	%OH	141.666	2.221	−3.23
				MET1178	%NH-pyrrole	#CONH	156.864	2.028	−4.51
hK_v_11.1	R-TGB	−5.4	3.32	none					
	S-TGB	−5.2	3.14	TYR652	%NH	#CONH	136.087	1.97	−2.86
				SER660	#OH	%COO	128.896	2.145	−0.04
	NFD	−4.42	3.24	ASN658	#CONH_2_	%NO	174.448	1.849	−7.63
	R-TEF	−8.4	6.27	TYR652	%OH	#PhOH	158.395	2.121	−3.63
	S-TEF	−8.4	6,56	none					
	BTX	−6.75	4.95	PHE551	%OH	#CONH	171.728	1.933	−0.28
				THR623			140.298	1.933	−2.24

Abbreviations in Table. Components of the investigated complexes: protein-hNav1.5, hCav1.2 and hK_v_11.1 (hERG); and ligands, R/S-TGB (R/S-tiagabine), NFD (nifedipine), R/S-TEF (R/S-terfenadine), BTX (batrachotoxin). Other abbreviations: H_B_—hydrogen bond. acc—hydrogen bond acceptor. Hydrogen bond components: from the ligand % and from the protein #. E_B_—complex energy binding. θ—hydrogen bond angle. L_HB_—hydrogen bond length. E_HB_—hydrogen bond energy. pK_i_ was calculated from the AutoDock4 and estimated inhibition constant K_i_, which is reported in the AutoDock4 output.

**Table 3:** The summary of validation experiment results.

Complex		E_B_	pKi	Amino Acid Residues	H_B_		Angle	L_HB_	E_HB_
Protein	Ligand				donor	acc	θ	Å	kcal/mol
hNa_v_1.5	PRG	−3.90	2.86	ASP945	%CONH1	#COO	157.949	1.998	−5.425
				ASN1474	%CONH2	#CO	155.21	2.00	−5.04
				ASN1474	#NH2	%CO	141.795	1.987	−2.731
	ASA	−3.29	2.41	LYS1477	#NH1	%CO	159.455	1.905	−2.071
				LYS1477	#NH2	%COO	157.938	1.737	−0.015
hCa_v_1.2	PRG	−3.29	2.41	THR1056	%OH	#OH	163.88	1.99	−4.82
				SER1132	#OH	%CONH	156.74	1.94	−3.02
	ASA	−2.41	1.77	THR1056	#OH	%COO	159.14	1.84	−2.30
				SER1132	#OH	%CO	176.21	1.945	−6.00
hK_v_11.1	PRG	−3.4	2.50	TYR652	%CONH2	#PhOH	139.884	1.902	−2.015
				TYR652	%NH	#PhOH	139.121	2.182	−1.695
				SER660	%OH	#OH	170.052	2.129	−0.893
	ASA	−3.26	2.39	ASN658	#CONH2	%COO	167.92	1.78	−7.29

Abbreviations in Table. Components of the investigated complexes: protein-hNav1.5, hCav1.2 and hKv11.1 (hERG); and ligands, ASA—acetylsalicylic acid, PRG—Progabide. Other abbreviations: H_B_—hydrogen bond. acc—hydrogen bond acceptor. Hydrogen bond components: from the ligand % and from the protein #. E_B_—complex energy binding. θ—hydrogen bond angle. L_HB_—hydrogen bond length. E_HB_—hydrogen bond energy. pKi was calculated from the AutoDock4 and estimated inhibition constant K_i_, which is reported in the AutoDock4 output [55].

**Table 4:** The composition of the binding pocket of the analyzed channel models: hNav1.5, hCav1.2 and hKv11.1.

Protein	Intramembrane Pore-Forming Region Sequences
hNav1.5	358–382 (Phe, Ala, Trp, Ala, Phe, Leu, Ala, Leu, Phe, Arg, Leu, Met, Thr, Gly, Leu, Ans, Asp, Cys, Trp, Glu, Arg, Leu, Tyr, Gly, Leu, Ans, Gly, Leu, Ans, Thr, Leu) 884–904 (Phe, Phe, His, Ala, Phe, Leu, Ile, Ile, Phe, Arg, Ile, Leu, Cys, Gly, Glu, Trp, Ile, Glu, Thr, Met, Trp) 1406–1427 (Gly, Ala, Gly, Tyr, Leu, Ala, Leu, Leu, Gly, Leu, Ans, Val, Ala, Thr, Phe, Lys, Gly, Trp, Met, Asp, Ile, Met, Tyr, Ala) 1697–1719 (Phe, Ala, Ans, Ser, Met, Leu, Cys, Leu, Phe, Gly, Leu, Ans, Ile, Thr, Thr, Ser, Ala, Gly, Trp, Asp, Gly, Leu, Leu, Ser, Pro) [84,85]
hCav1.2	351–372 (Phe, Ala, Met, Leu, Thr, Val, Phe, Gly, Leu, Ans, Cys, Ile, Thr, Met, Glu, Glu, Trp, Thr, Asp, Val, Leu, Tyr, Trp, Val) 694–715 (Gly, Leu, Ans, Ser, Leu, Leu, Thr, Val, Phe, Gly, Leu, Ans, Ile, Leu, Thr, Gly, Glu, Asp, Trp, Ans, Ser, Val, Met, Tyr, Asp, Gly) 1122–1142 (Leu, Ala, Ala, Met, Met, Ala, Leu, Phe, Thr, Val, Ser, Thr, Phe, Glu, Gly, Trp, Pro, Glu, Leu, Leu, Tyr) 1453–1471 (Ala, Val, Leu, Leu, Leu, Phe, Arg, Cys, Ala, Thr, Gly, Glu, Ala, Trp, Gly, Leu, Ans, Asp, Ile, Met, Leu) [86,87]
hKv11.1	612–632 (Val, Thr, Ala, Leu, Tyr, Phe, Tphe, Ser, Ser, Leu, Thr, Ser, Val, Gly, Phe, Gly, Ans, Vsp) [88,89]

## Data Availability

Not applicable.

## Supplementary Materials

The following are available online, Figure S1: Model transport proteins selected for the study: hNav1.5, hCav1.2, Kv11.1. Figure S2: Figure S3: Binding modes between hNav1.5, hCav1.2 and Kv11.1 and the four tested compounds () and two validated compounds

## Sample Availability

Samples of the compounds are not available from the authors.

## Abbreviations

AP	Cardiac action potential
(R/S)-TGB	(R/S)-Tiagabine
(R/S)-TEF	((R/S)-Terfenadine
NFD	Nifedipine
BTX	Batrachotoxin
#	Hydrogen bond components: from the protein
%	Hydrogen bond components: from the ligand
Acc	Hydrogen bond acceptor
EB	Complex energy binding
EHB	Hydrogen bond energy
θ	Hydrogen bond angle
VGICs	Voltage-Gated Ion Channels
VGKCs	Voltage-Gated Potassium. Channels
VGNaCs	Voltage-Gated Sodium Channels
VGCaCs	Voltage-Gated Calcium Channels
KV11.1 (hERG)	Cardiac Voltage-Gated Potassium Channels
NaV1.5	Cardiac Voltage-Gated Sodium Channels
CaV1.2	Cardiac Voltage-Gated Calcium Channels
