# Supplementary material for: Antiepileptic Drug Tiagabine Does Not Directly Target Key Cardiac Ion Channels Kv11.1, Nav1.5 and Cav1.2
Source: Molecules. 2021 Jun 9;26(12):3522. doi: 10.3390/molecules26123522 (PMC8226520; doi:10.3390/molecules26123522)
Supplement: Supplementary file 1 [file molecules-26-03522-s001.zip › molecules-1213614-SI V1-.pdf]

molecules

Supplementary Material

Type of the Paper (Article)

Role of voltage-gated ion channels in tiagabine-induced long-QT related arrhythmias

Magdalena Kowalska[1], Łukasz Fijałkowski[1], Monika Kubacka[2], Kinga Sałat[2], Grzegorz Grześk[3], Jacek Nowaczyk[4], Alicja Nowaczyk[1]*

[1] Department of Organic Chemistry, Faculty of Pharmacy, Ludwik Rydygier Collegium Medicum in Bydgoszcz, Nicolaus Copernicus University in Toruń, Poland; M.K.: magda.kowalska@doktorant.umk.pl ; Ł.F.: l.fijalkowski@cm.umk.pl ; A.N.: alicja@cm.umk.pl

[2] Department of Pharmacodynamics, Chair of Pharmacodynamics, Jagiellonian University Medical College, 9 Medyczna St., 30 - 688 Krakow, Poland; K.S. kinga.salat@uj.edu.pl, M.K. monika.kubacka@ uj.edu.pl

[3] Department of Cardiology and Clinical Pharmacology, Faculty of Health Sciences, Ludwik Rydygier Collegium Medicum in Bydgoszcz, Nicolaus Copernicus University, 75 Ujejskiego St., 85-168 Bydgoszcz, Poland; G.G. g.grzesk@cm.umk.pl

[4] Chair of Physical Chemistry and Chemistry of Polymers, Faculty of Chemistry, Nicolaus Copernicus University, 7 Gagarina St, 87-100 Toruń, Poland; J.N. jacek.nowaczyk@umk.pl

* Correspondence: alicja@cm.umk.pl, ORCID: 0000-0003-4945-2369;

E-mail address and ORCID for all of the co-authors :

Magdalena Kowalska: magda.kowalska@doktorant.umk.pl ORCID: 0000-0001-7950-5543
Łukasz Fijałkowski: l.fijalkowski@cm.umk.pl ORCID: 0000-0001-6778-8259
Monika Kubacka: monika.kubacka@uj.edu.pl ORCID: 0000-0003-4878-0723
Kinga Sałat: kinga.salat@uj.edu.pl ORCID: 0000-0003-0614-5393
Grzegorz Grześk: g.grzesk@cm.umk.pl ORCID : 0000-0001-6669-5931
Jacek Nowaczyk: jacek.nowaczyk@umk.pl ORCID: 0000-0003-4521-8065
Alicja Nowaczyk: alicja@cm.umk.pl ORCID: 0000-0003-4945-2369

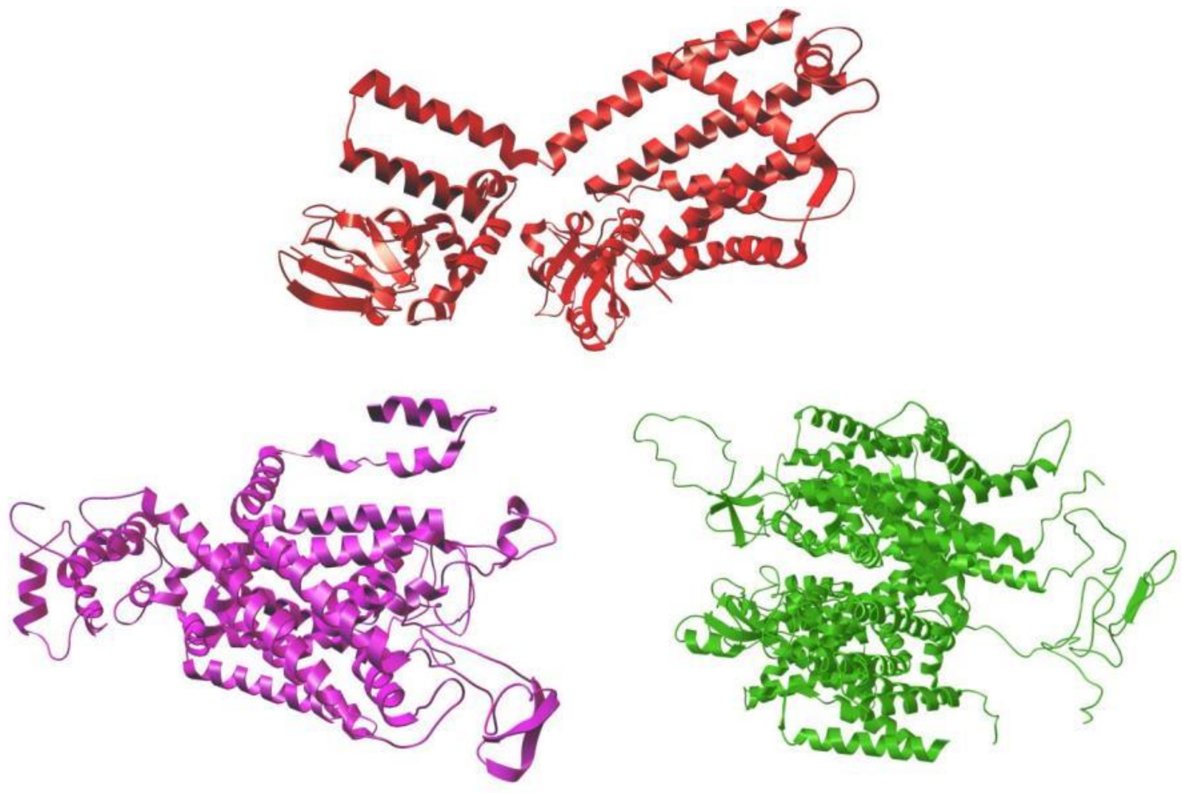

Figure S1. Protein models used in docking experiments: $Na_v1.5$ (green), $Ca_v1.2$ (purple), $K_v11.1$ (red)

Figure S2. **Molecular docking experiment:** Binding modes of (S)-Tiagabine ((R/S)-TGB). (R/S)-TEF ((R/S)-Terfenadine). NFD (Nifedipine). BTX (Batrachotoxin) to hNav1.5, hCav1.2 and hKv11.1 channels; Ligands (ball and stick model), calculated hydrogen bonds (dashed green lines).

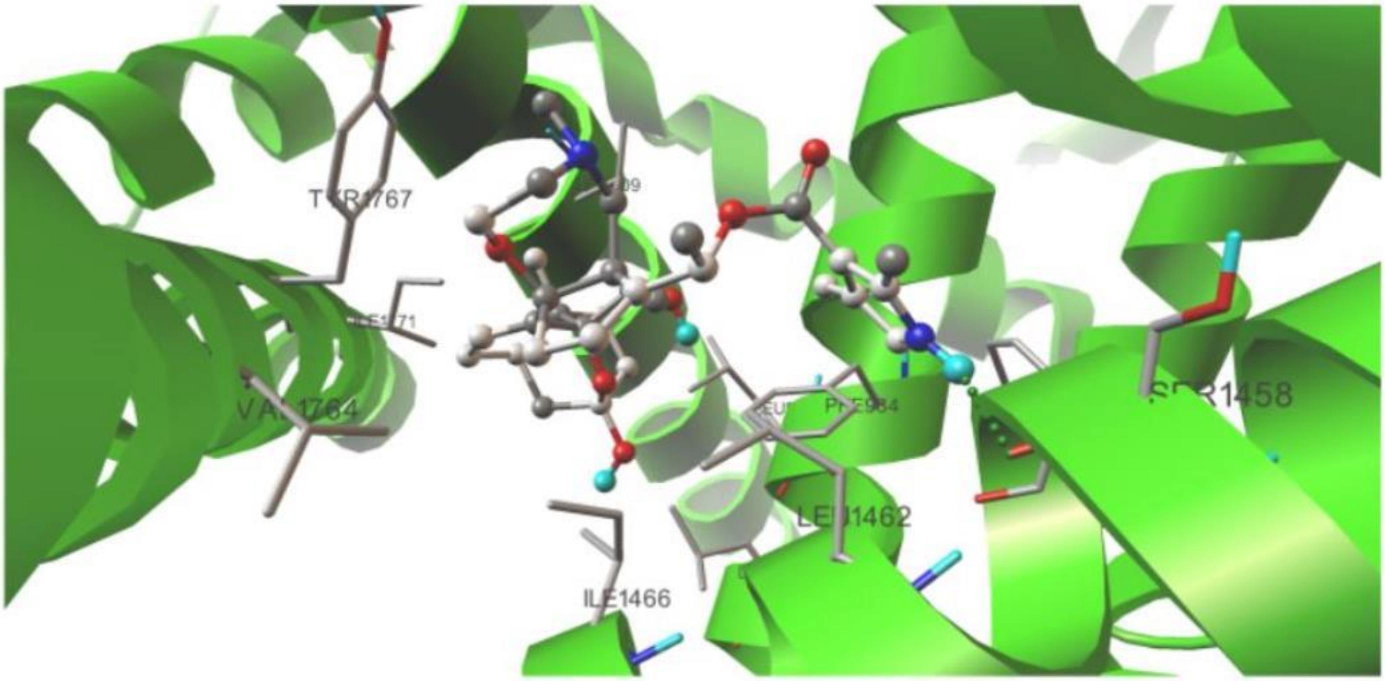

Figure S2. *Binding modes between* BTX (Batrachotoxin) to hNav1.5. Ligands (ball and stick model), calculated hydrogen bonds (dashed green lines).

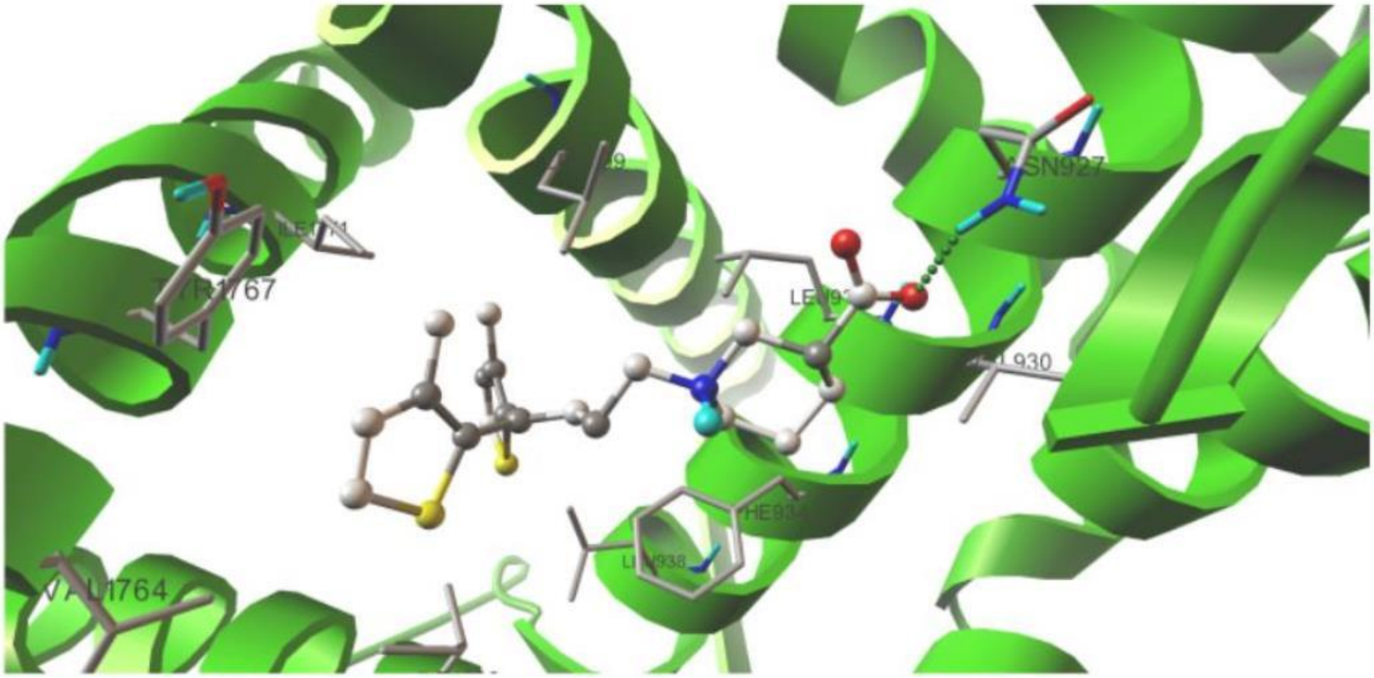

Figure S2. *Binding modes between* (R)-Tiagabine ((R)-TGB) to hNav1.5. Ligands (ball and stick model), calculated hydrogen bonds (dashed green lines).

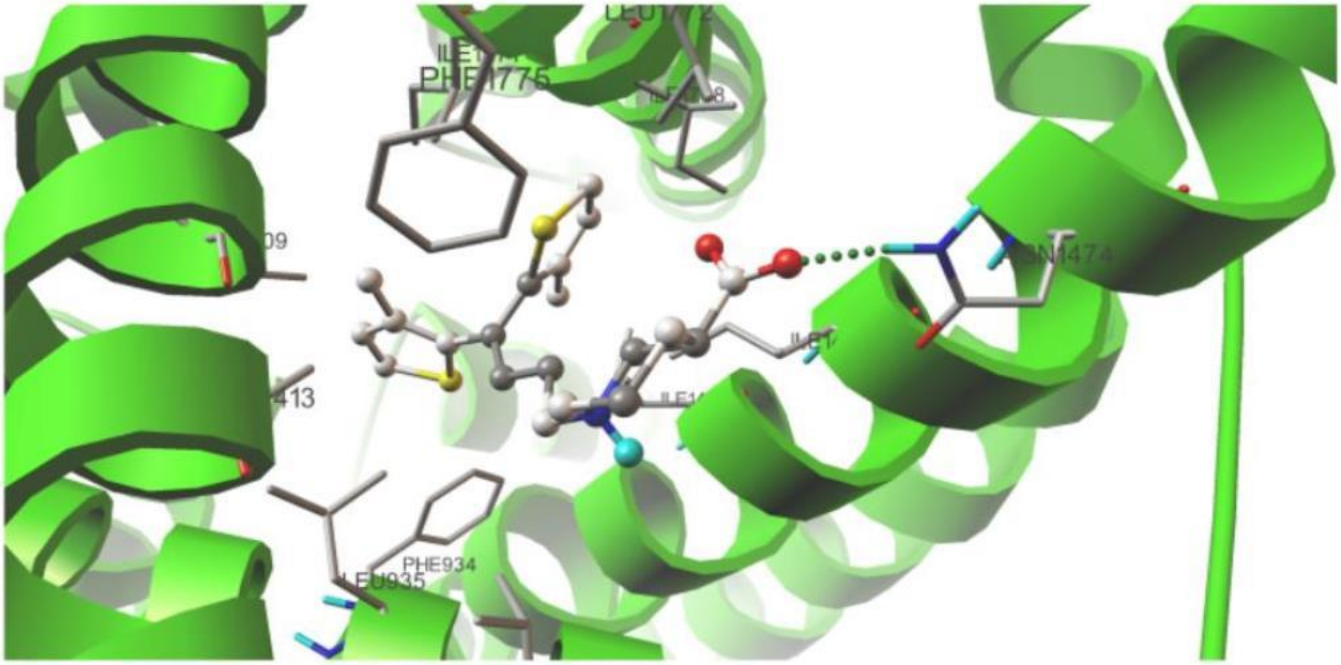

Figure S2. *Binding modes between* (S)-Tiagabine ((S)-TGB) to hNav1.5. Ligands (ball and stick model), calculated hydrogen bonds (dashed green lines).

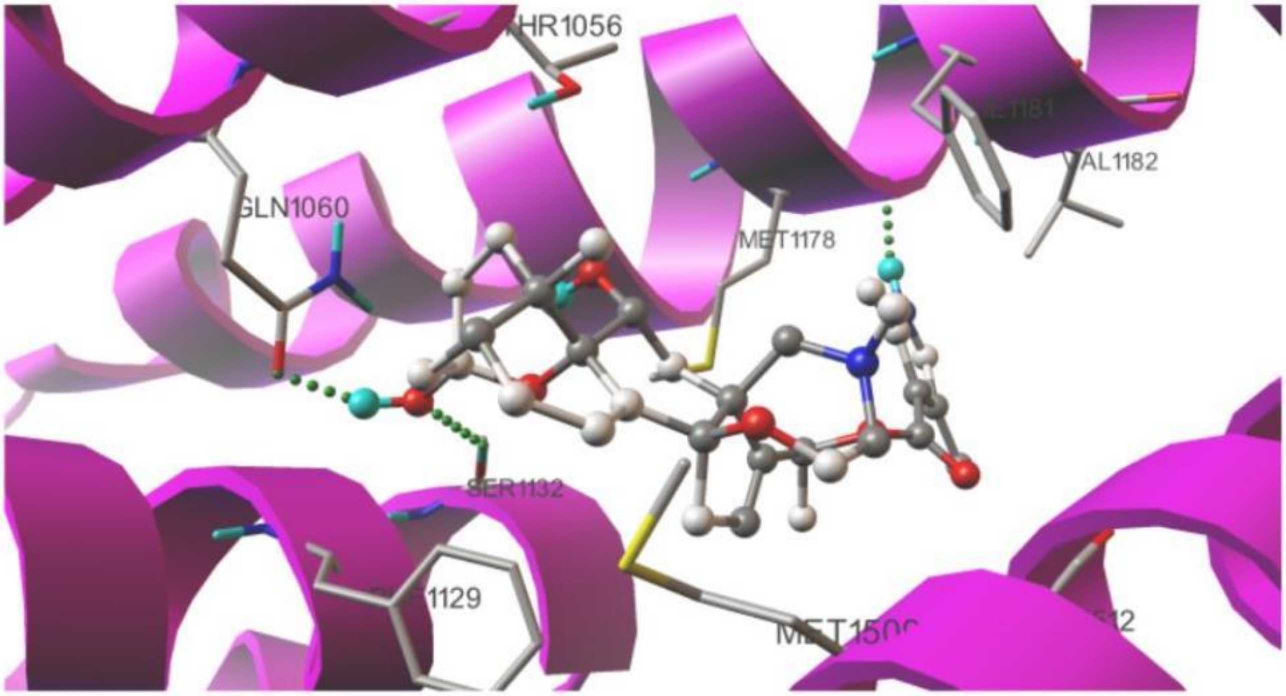

Figure S2. Binding modes between BTX (Batrachotoxin) to hCav1.2. Ligands (ball and stick model), calculated hydrogen bonds (dashed green lines).

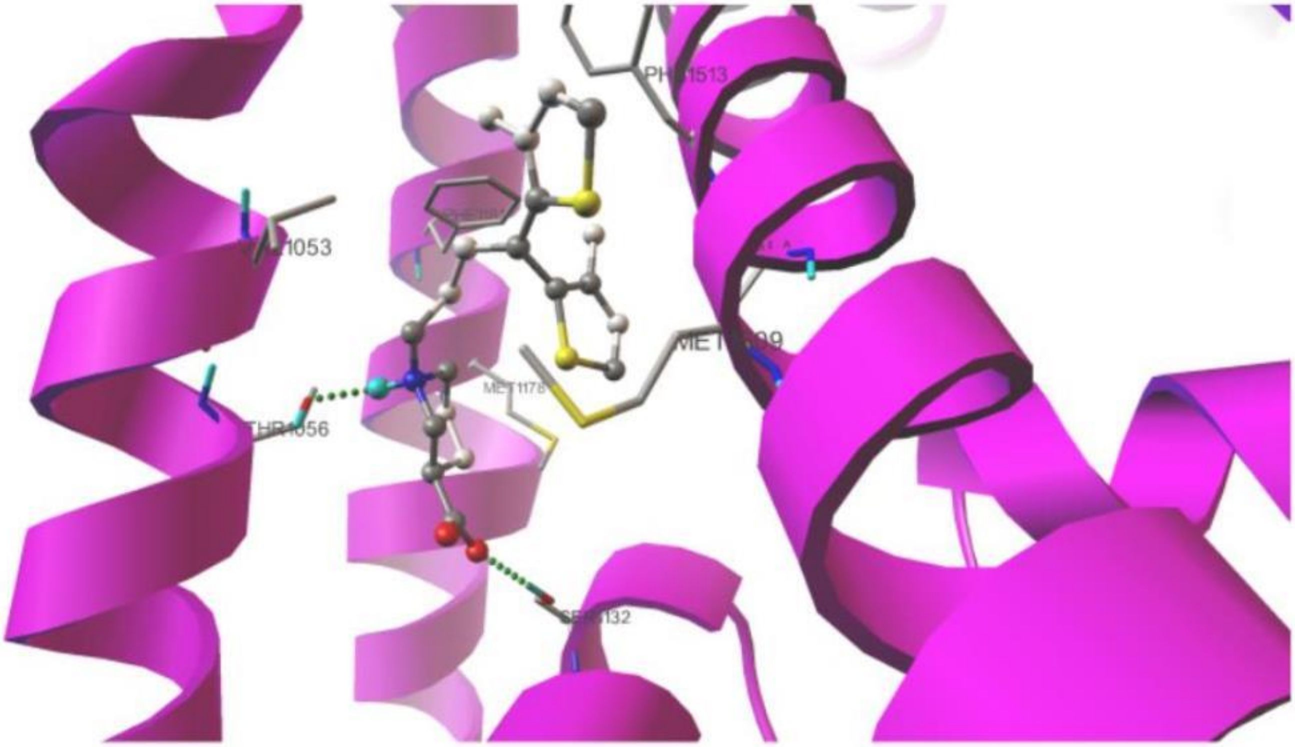

Figure S2. *Binding modes between* (R)-Tiagabine ((R)-TGB) to hCav1.2. Ligands (ball and stick model), calculated hydrogen bonds (dashed green lines).

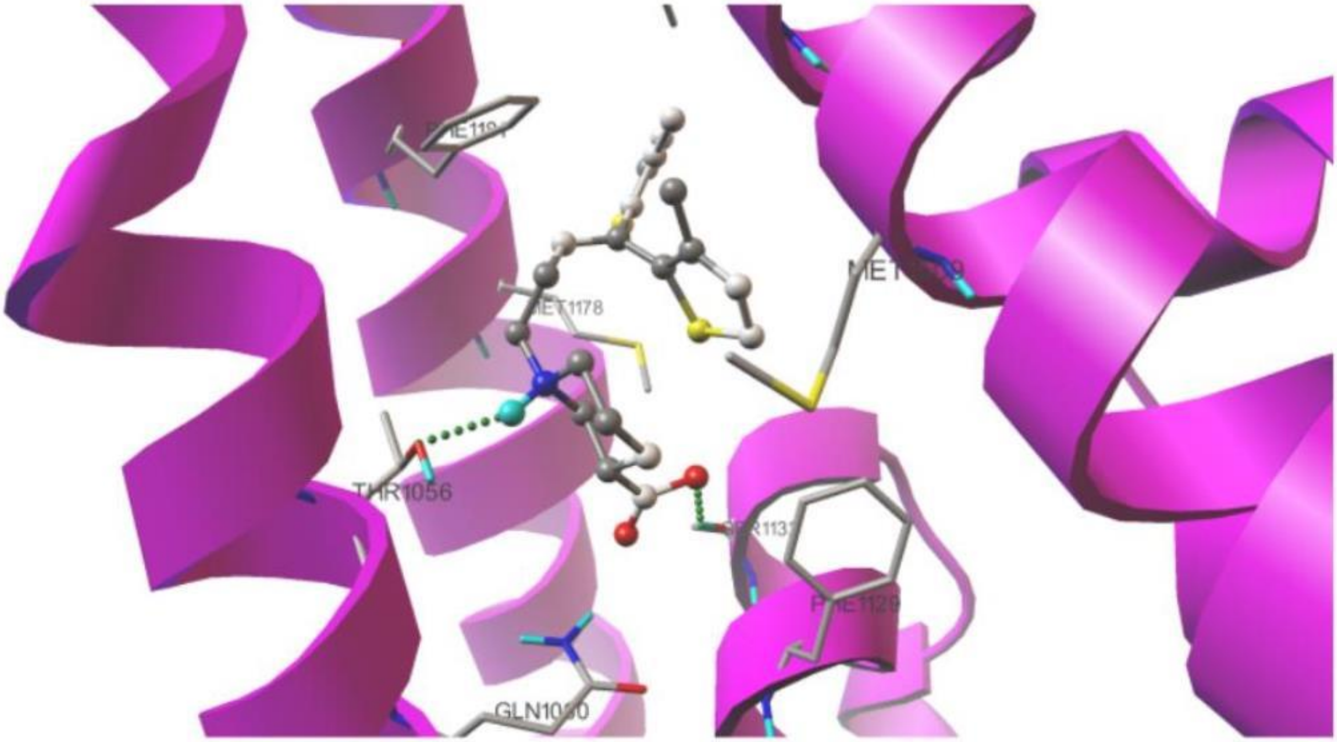

Figure S2. *Binding modes between* (S)-Tiagabine ((S)-TGB) to hCav1.2. Ligands (ball and stick model), calculated hydrogen bonds (dashed green lines).

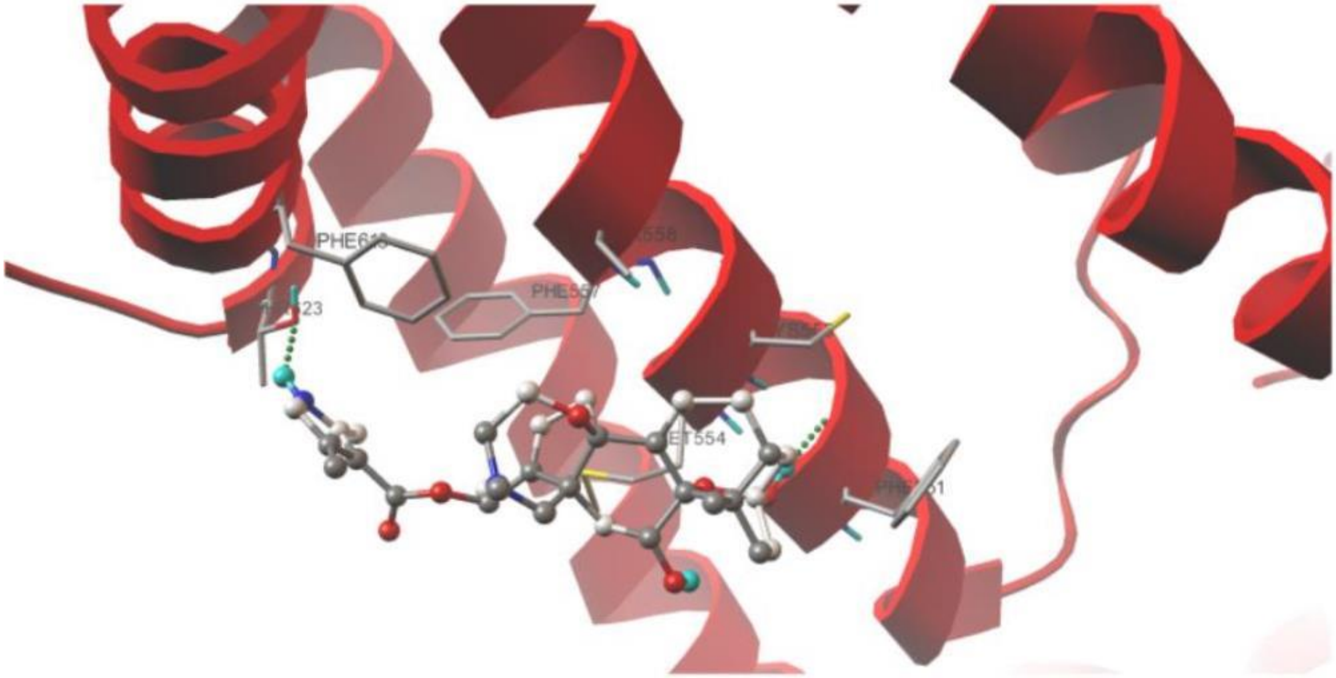

Figure S2. Binding modes between BTX (Batrachotoxin) to hKv11.1. Ligands (ball and stick model), calculated hydrogen bonds (dashed green lines).

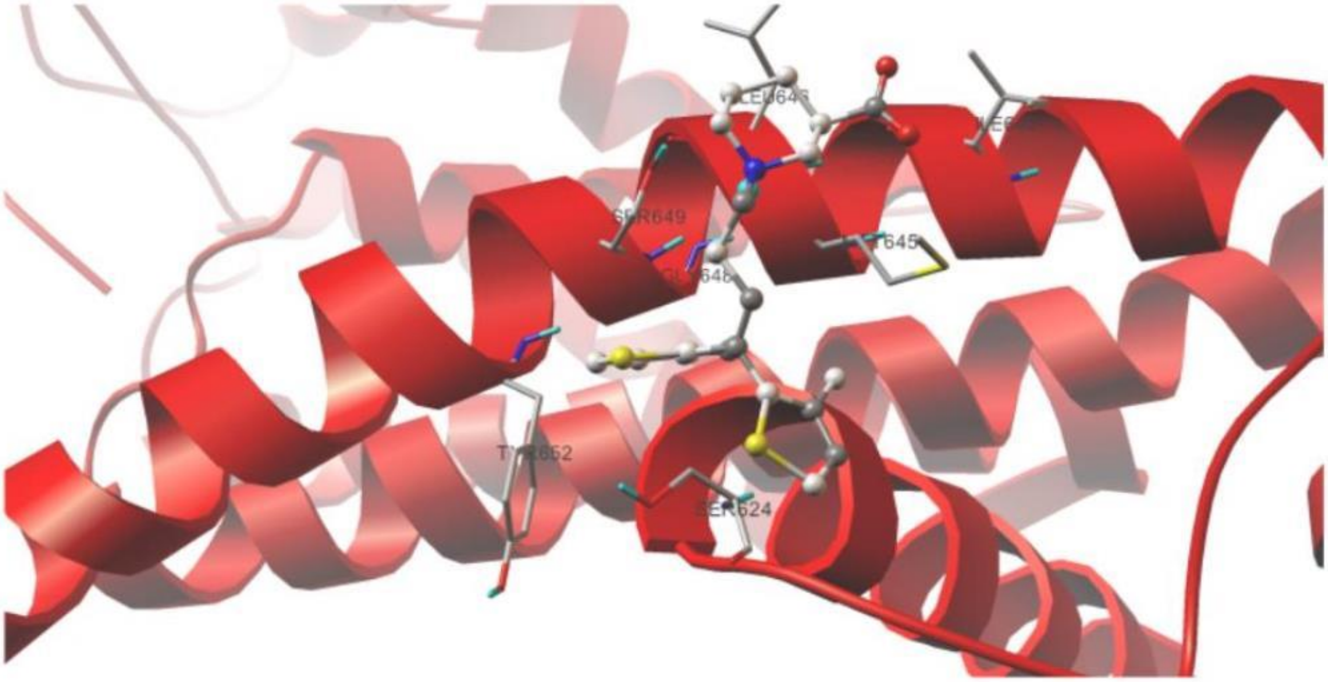

Figure S2. *Binding modes between* (R)-Tiagabine ((R)-TGB) to hKv11.1. Ligands (ball and stick model), calculated hydrogen bonds (dashed green lines).

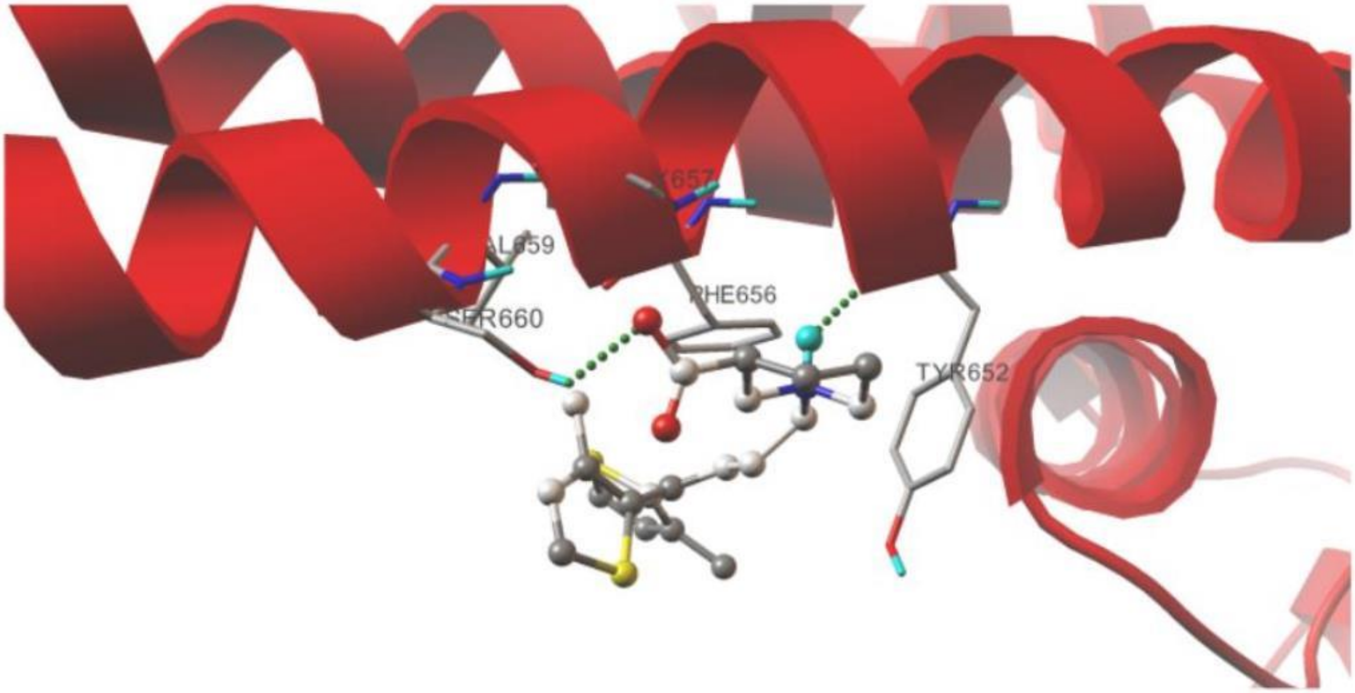

Figure S2. *Binding modes between* (S)-Tiagabine ((S)-TGB) to hKv11.1. Ligands (ball and stick model), calculated hydrogen bonds (dashed green lines).

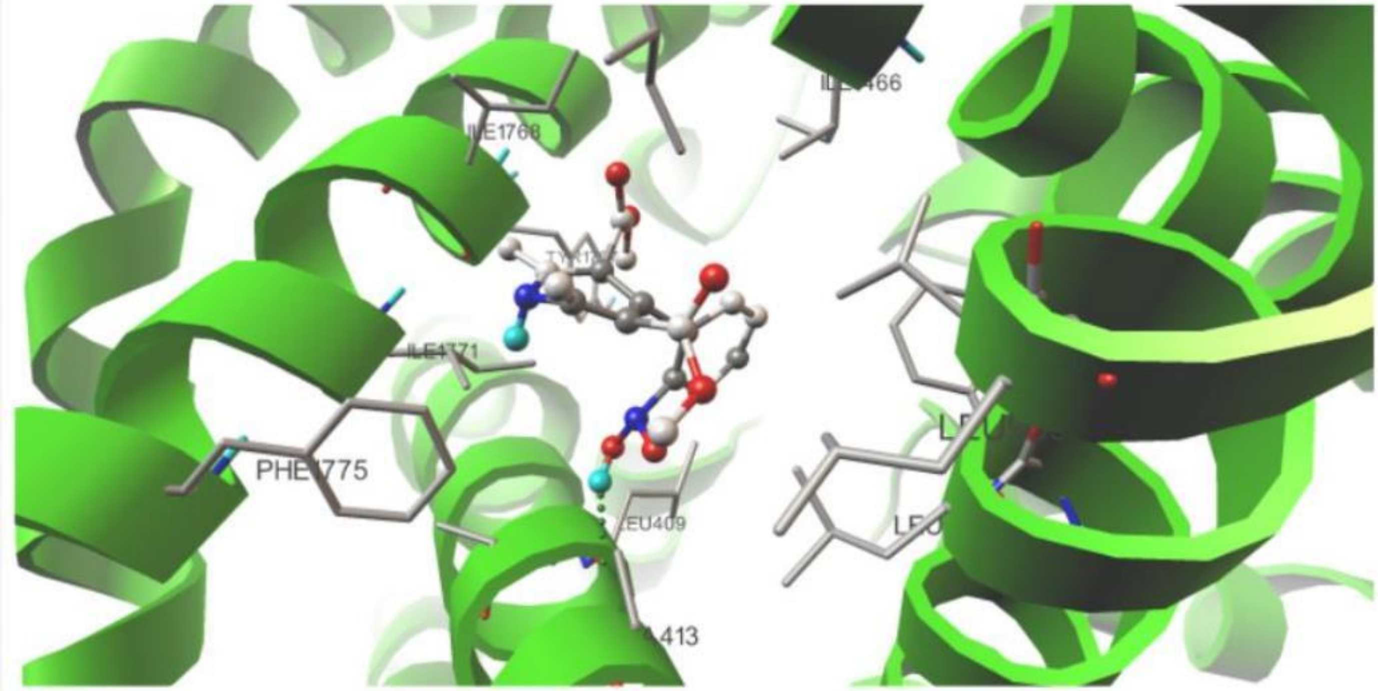

Figure S2. *Binding modes between* NFD (Nifedipine) to hNav1.5. Ligands (ball and stick model), calculated hydrogen bonds (dashed green lines).

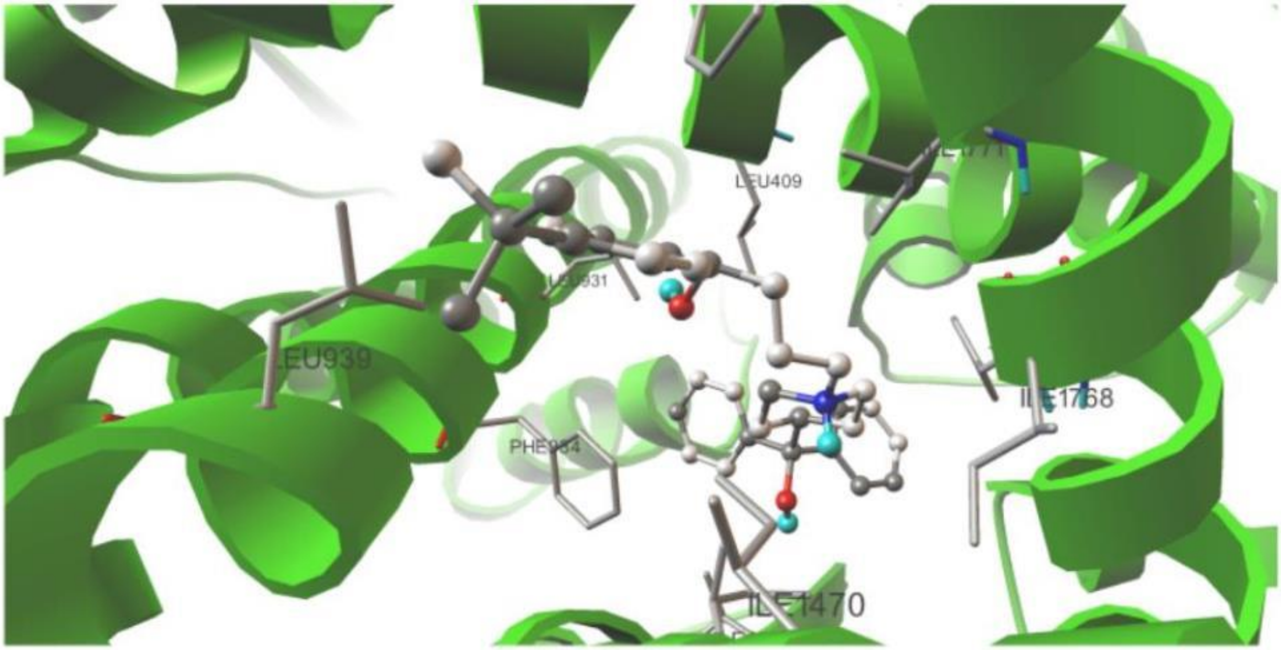

Figure S2. *Binding modes between* (R)-TEF ((R)-Terfenadine) to hNav1.5. Ligands (ball and stick model), calculated hydrogen bonds (dashed green lines).

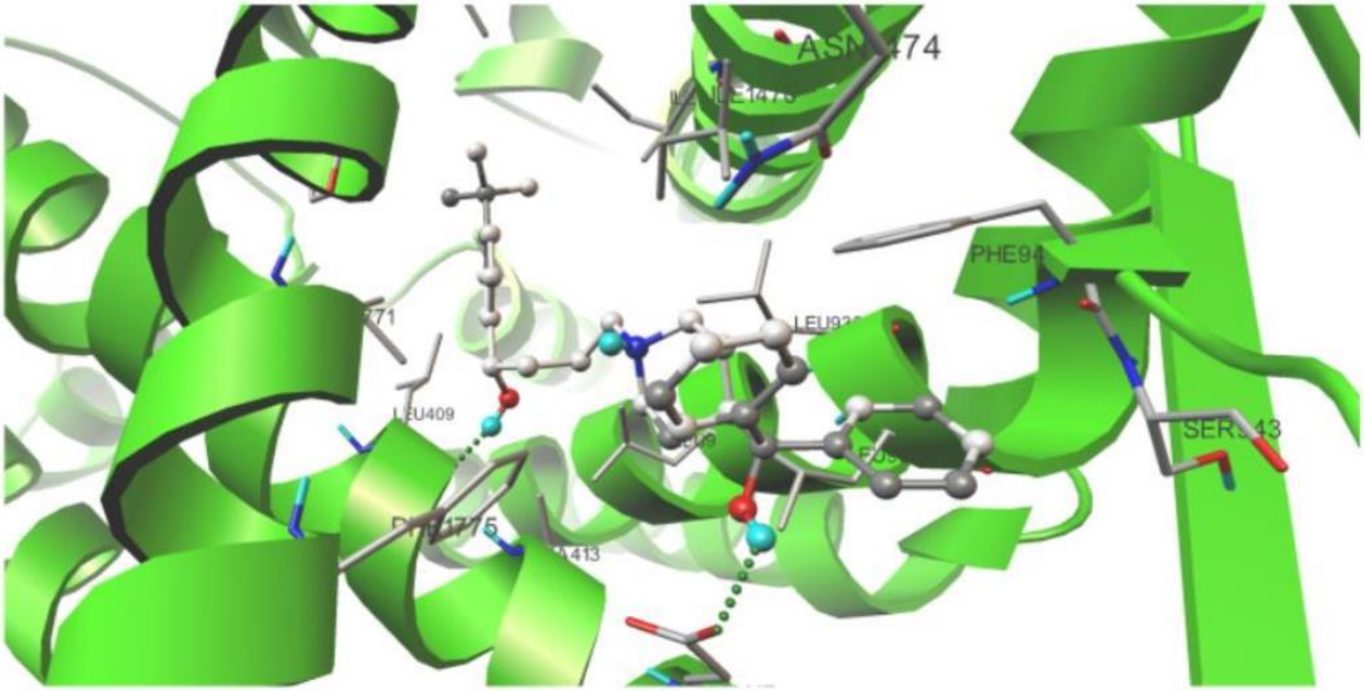

Figure S2. *Binding modes between* (S)-TEF ((S)-Terfenadine) to hNav1.5. Ligands (ball and stick model), calculated hydrogen bonds (dashed green lines).

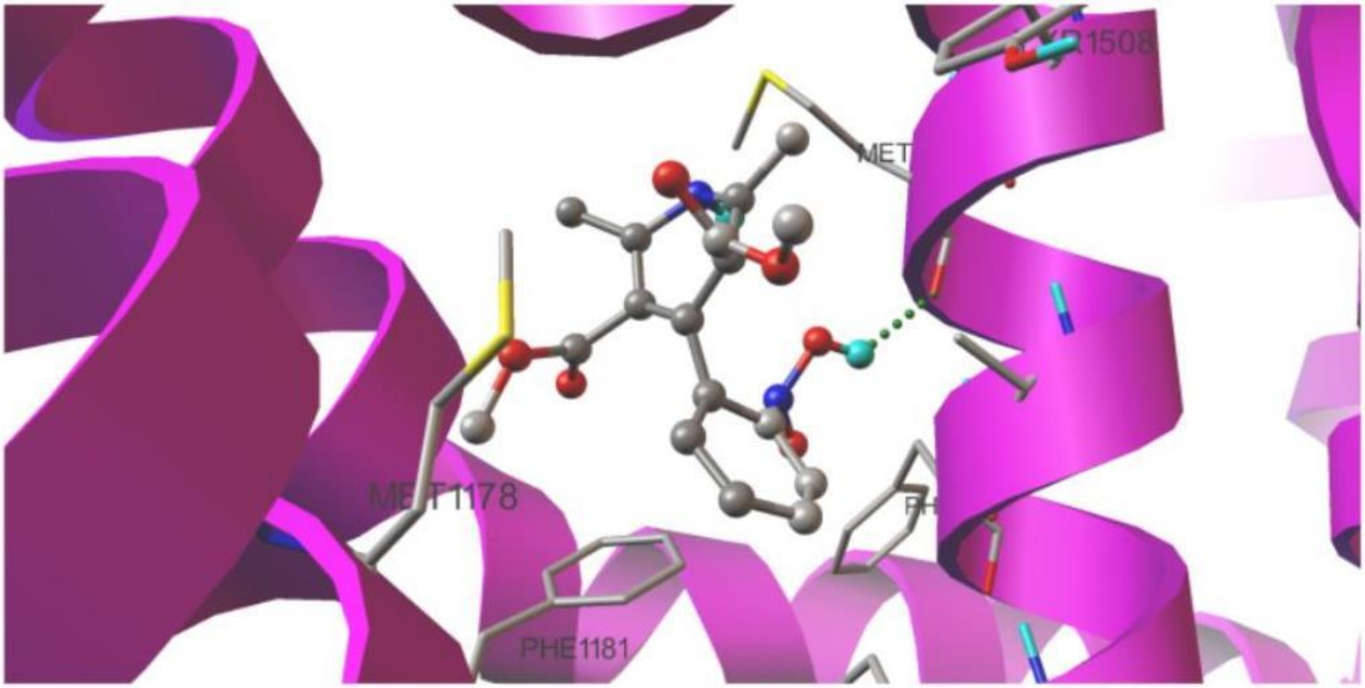

Figure S2. *Binding modes between* NFD (Nifedipine) to hCav1.2. Ligands (ball and stick model), calculated hydrogen bonds (dashed green lines).

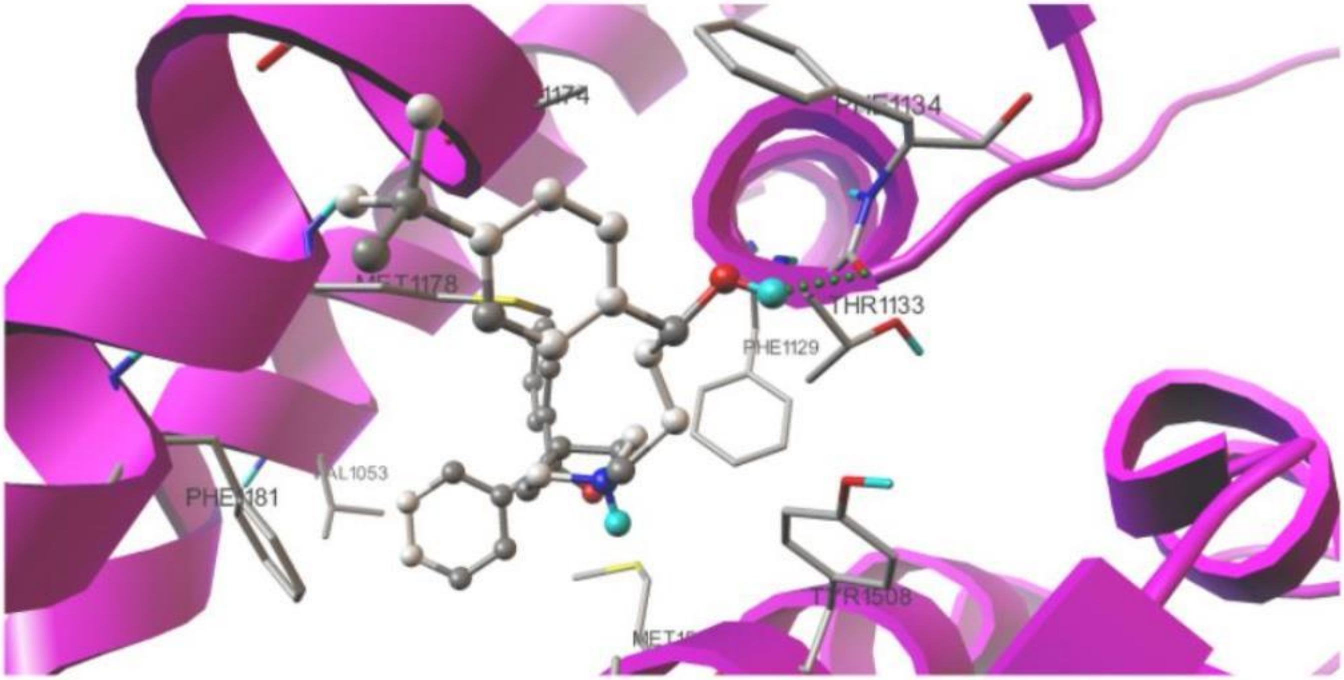

Figure S2. *Binding modes between* (R)-TEF ((R)-Terfenadine) to hCav1.2. Ligands (ball and stick model), calculated hydrogen bonds (dashed green lines).

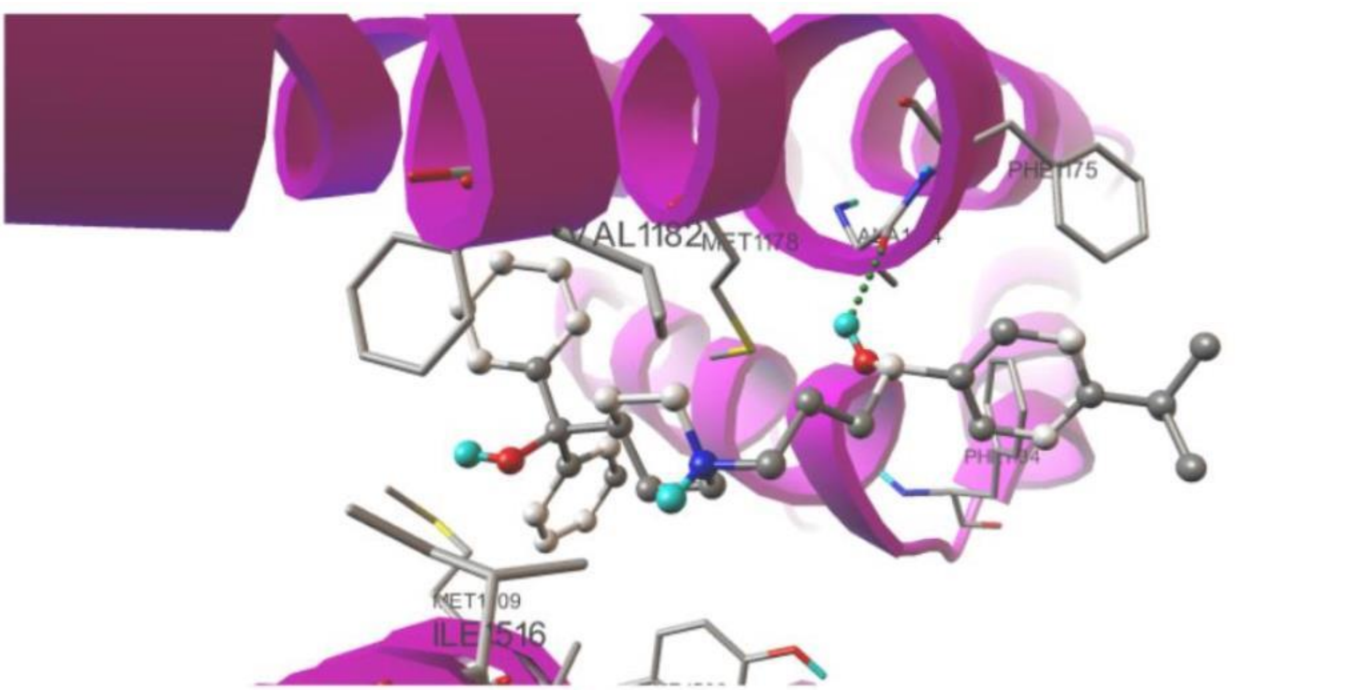

Figure S2. *Binding modes between* (S)-TEF ((S)-Terfenadine) to hCav1.2. Ligands (ball and stick model), calculated hydrogen bonds (dashed green lines).

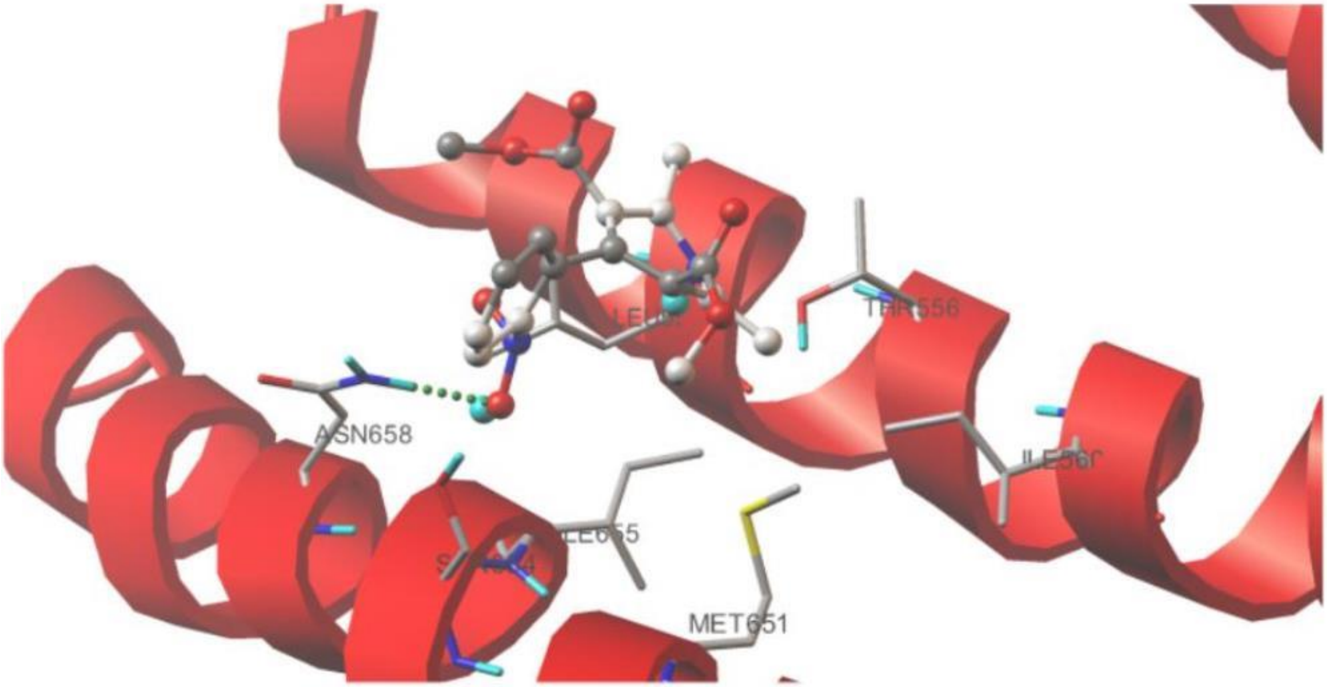

Figure S2. *Binding modes between* NFD (Nifedipine) to hKv11.1. Ligands (ball and stick model), calculated hydrogen bonds (dashed green lines).

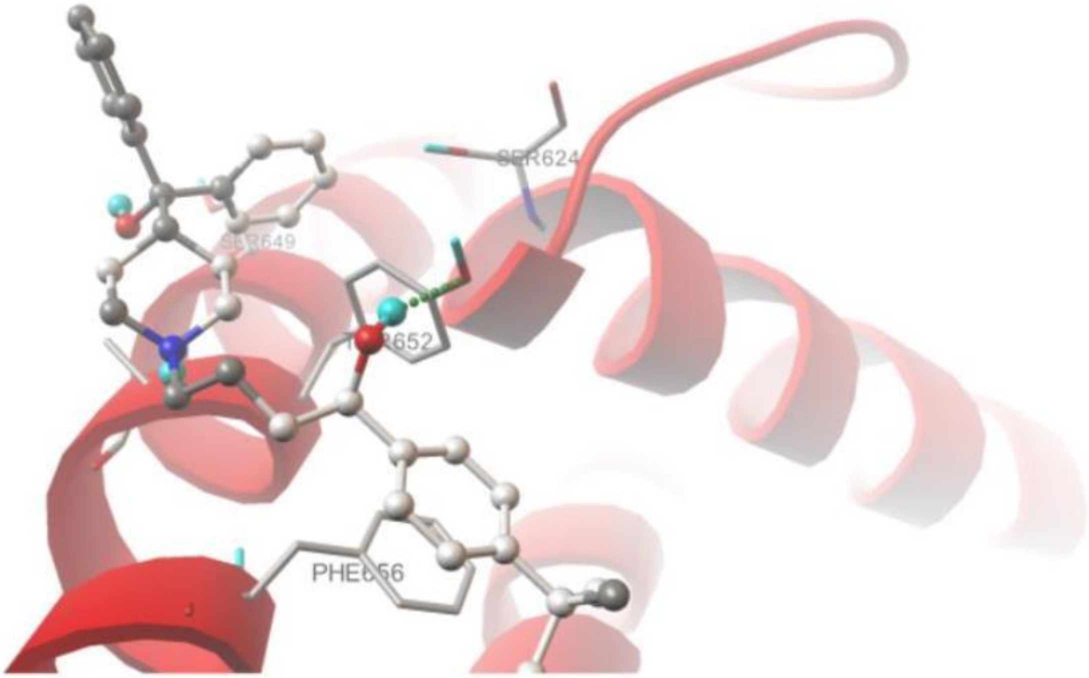

Figure S2. *Binding modes between* (R)-TEF ((R)-Terfenadine) to hKv11.1. Ligands (ball and stick model), calculated hydrogen bonds (dashed green lines).

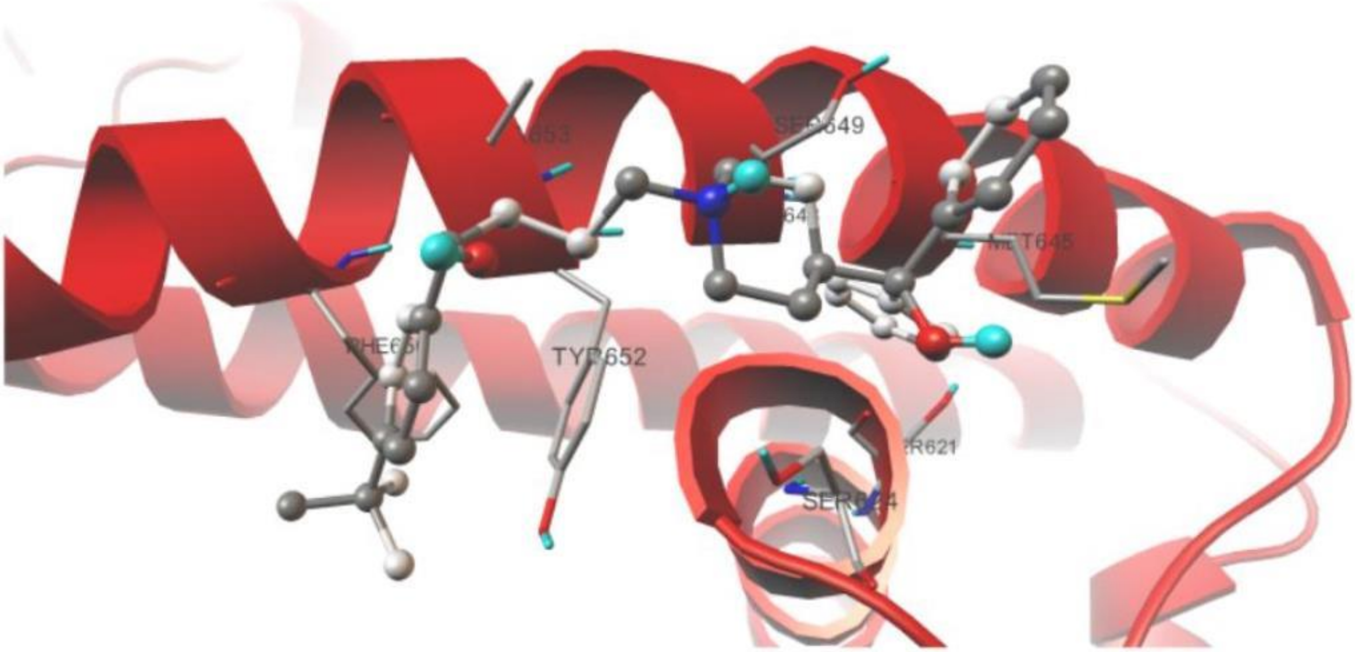

Figure S2. *Binding modes between* (S)-TEF ((S)-Terfenadine) to hKv11.1. Ligands (ball and stick model), calculated hydrogen bonds (dashed green lines).

Figure S3. Validation experiment: Binding modes of Progabide (PRG), Aspirin (ASA) to hNav1.5, hCav1.2 and hKv11.1 channels; Ligands (ball and stick model), calculated hydrogen bond

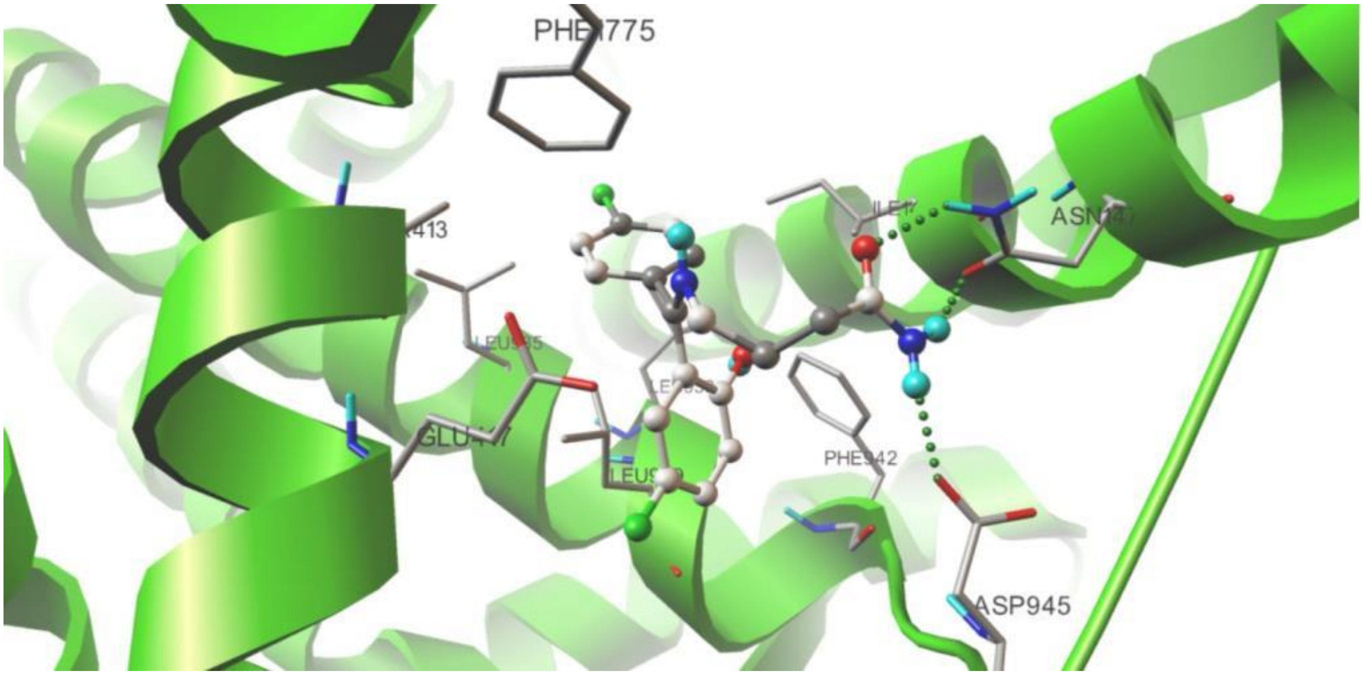

Figure S3. *Binding modes between* Progabide (PRG) to hNav1.5. Ligands (ball and stick model), calculated hydrogen bonds (dashed green lines).

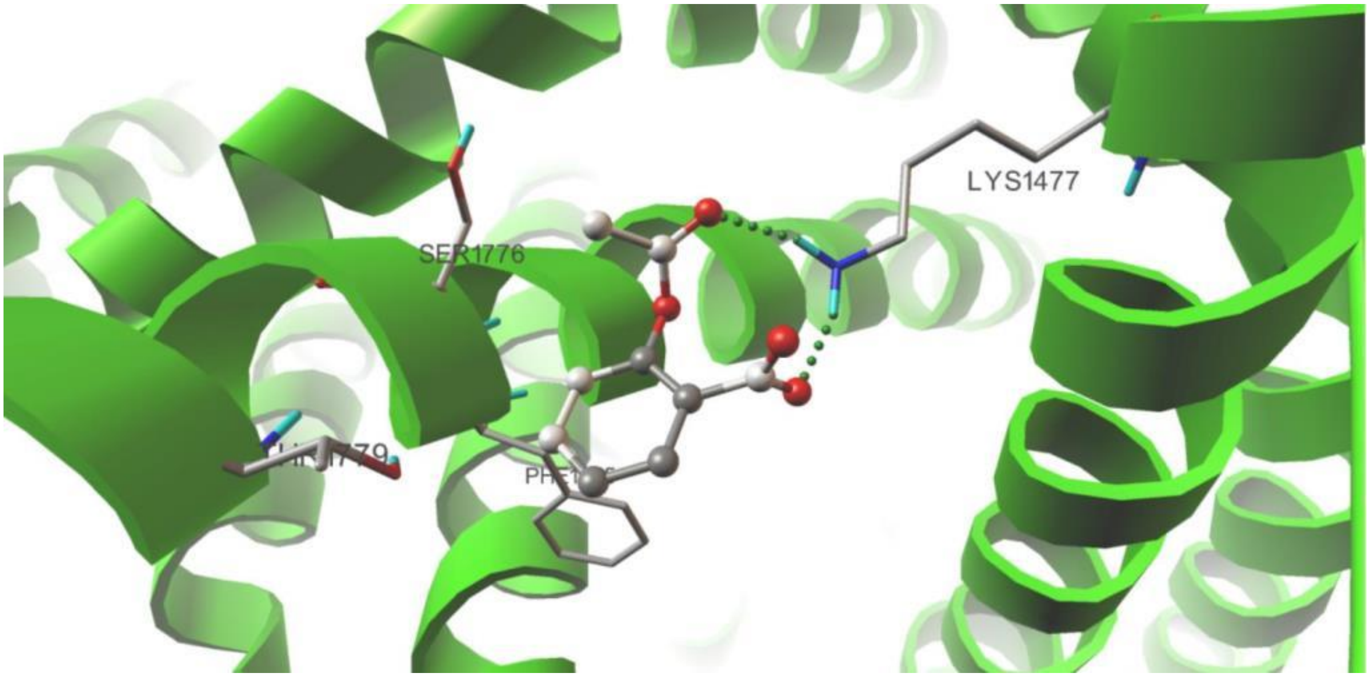

Figure S3. *Binding modes between* Aspirin (ASA) to hNav1.5. Ligands (ball and stick model), calculated hydrogen bonds (dashed green lines).

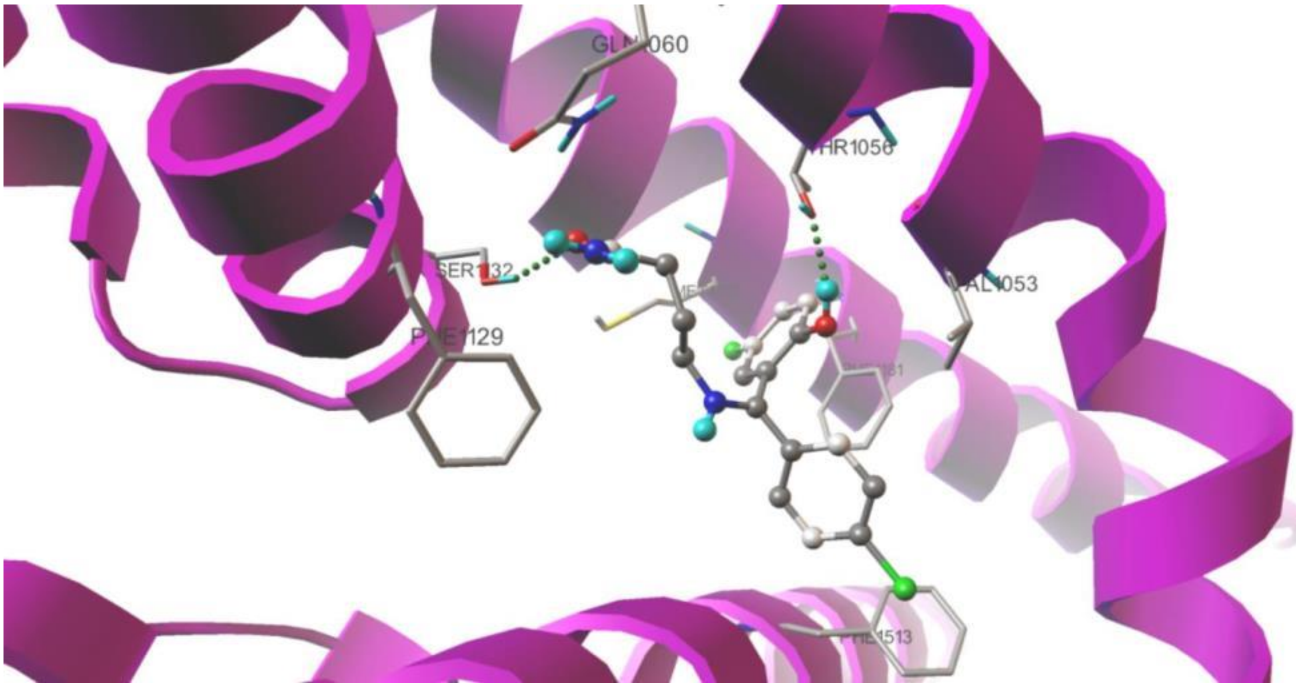

Figure S3. *Binding modes between* Progabide (PRG) to hCav1.2. Ligands (ball and stick model), calculated hydrogen bonds (dashed green lines).

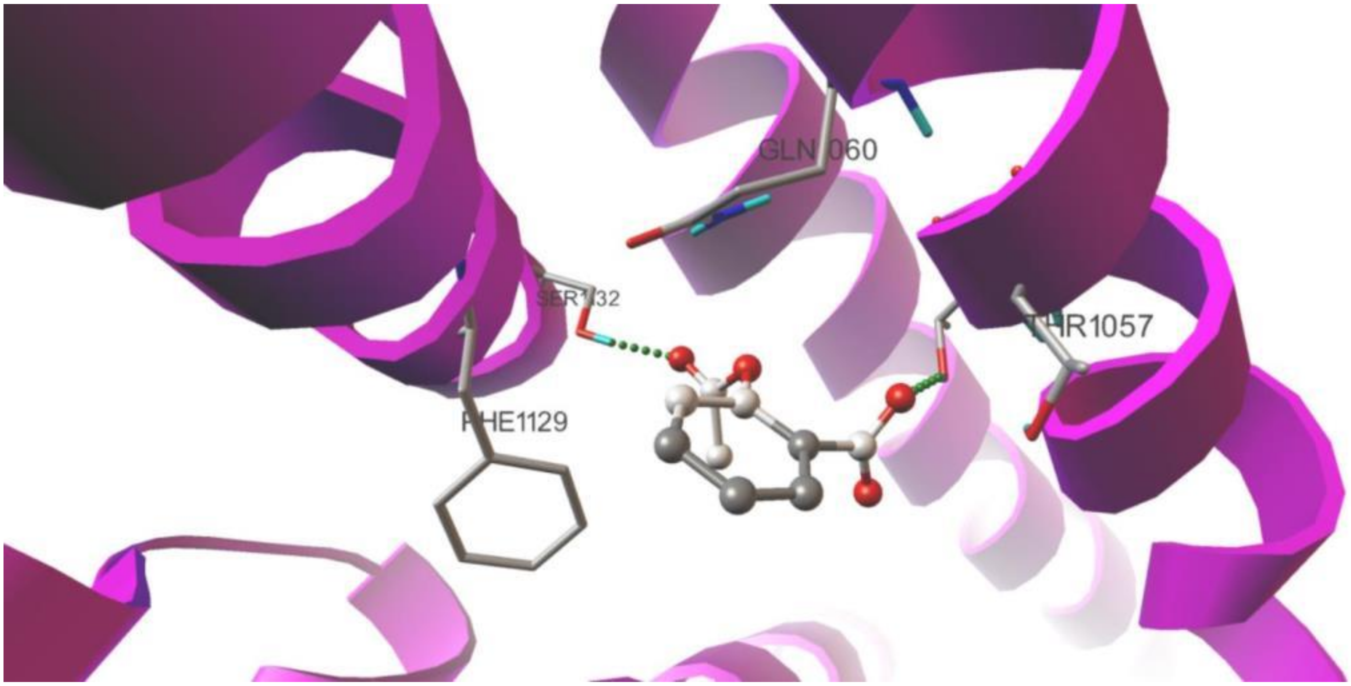

Figure S3. *Binding modes between* Aspirin (ASA) to hCav1.2. Ligands (ball and stick model), calculated hydrogen bonds (dashed green lines).

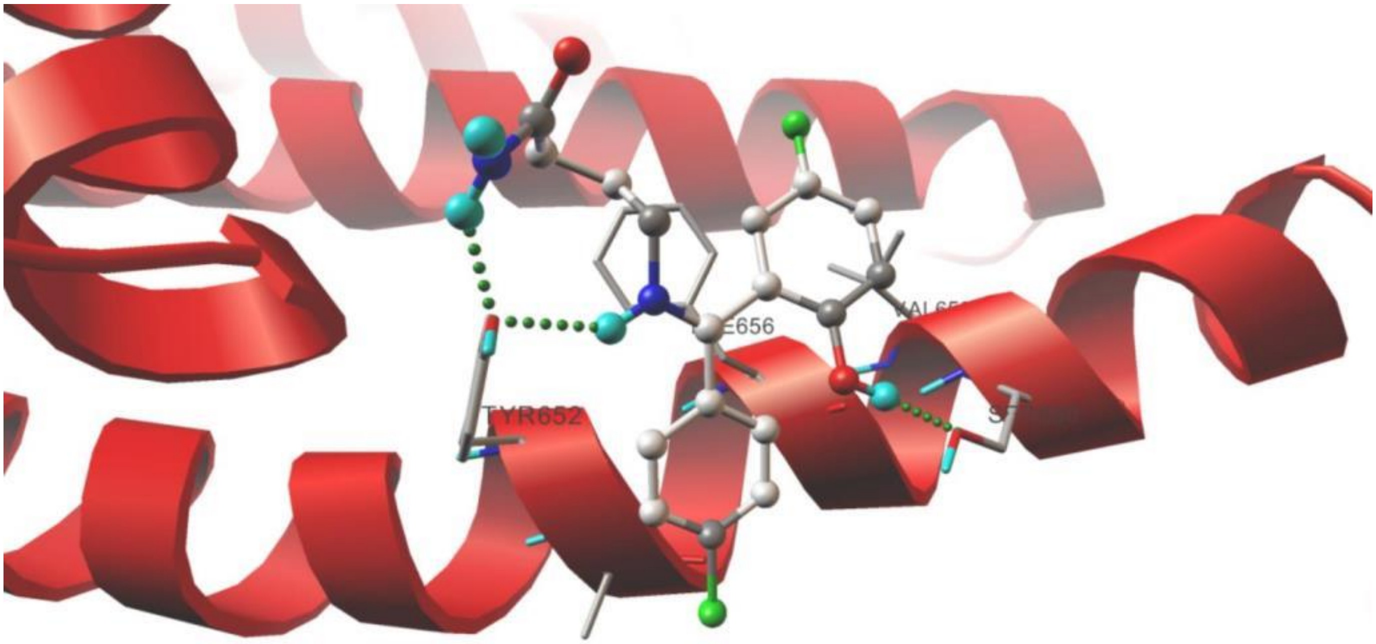

Figure S3. *Binding modes between* Progabide (PRG) to hKv11.1. Ligands (ball and stick model), calculated hydrogen bonds (dashed green lines).

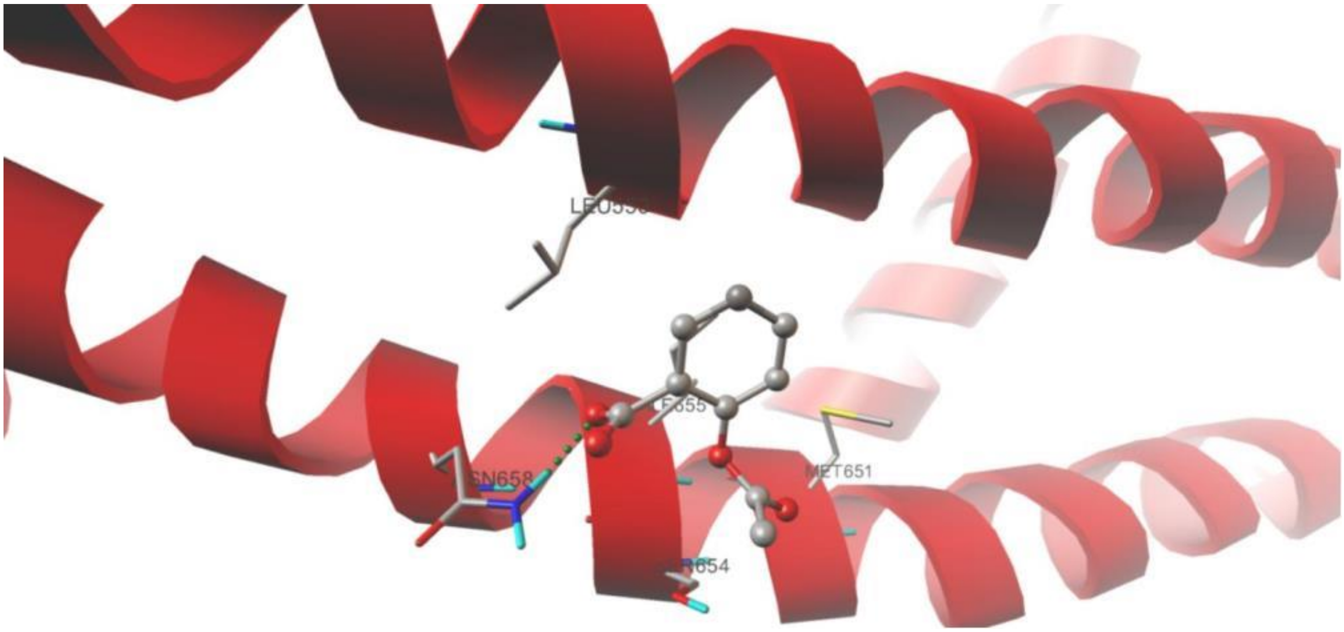

Figure S3. *Binding modes between* Aspirin (ASA) to hKv11.1. Ligands (ball and stick model), calculated hydrogen bonds (dashed green lines).
